# A mobile app-based intervention improves anthropometry, body composition and fitness, regardless of previous active-inactive status: a randomized controlled trial

**DOI:** 10.3389/fpubh.2024.1380621

**Published:** 2024-08-12

**Authors:** Nerea Gómez-Cuesta, Adrián Mateo-Orcajada, Lourdes Meroño, Lucía Abenza-Cano, Raquel Vaquero-Cristóbal

**Affiliations:** ^1^Facultad de Deporte, UCAM Universidad Católica de Murcia, Murcia, Spain; ^2^Research Group Movement Sciences and Sport (MS&SPORT), Department of Physical Activity and Sport, Faculty of Sport Sciences, University of Murcia, Murcia, Spain

**Keywords:** adolescents, anthropometry, body composition, gender, healthy lifestyle, mobile phone, mobile application, physical activity

## Abstract

**Introduction:**

The use of mobile apps to promote physical activity in adolescents can improve health-related parameters. However, previous studies have not evaluated whether the benefits depend on the users’ prior active or inactive status. Therefore, the main objective was to analyze differences in physical activity levels, adherence to the Mediterranean diet (AMD), anthropometry, body composition, and physical fitness between active and inactive adolescents.

**Methods:**

The study was conducted through a randomized controlled trial (RCT) with 462 adolescents, divided into experimental (EG) and control groups (CG), further categorized as active and inactive. Variables of physical activity, kinanthropometry, body composition, and physical fitness were measured before (pre-test) and after (post-test) a 10-week intervention using step-tracking apps (Strava, Pacer, MapMyWalk, and PokémonGo) at least three times per week.

**Results:**

The results showed that inactive EG adolescents significantly increased their physical activity levels, body mass, and muscle mass, and improved in all fitness variables except the countermovement jump (CMJ). The sum of three skinfolds also significantly decreased. Active EG adolescents increased body and muscle mass and improved in all fitness variables. Additionally, they significantly reduced fat mass and the sum of three skinfolds. All covariates, mainly gender and maturity, had significant effects on the study variables. Comparing changes between the active EG and CG groups, significant differences were found in body mass index (BMI) and CMJ in favor of the EG. However, while significant differences were observed in the study variables when analyzing each app individually, there were no differences between the changes produced by each app in these variables.

**Conclusion:**

After a 10-week program of physical activity promoted through step-tracking apps, improvements were observed in fat variables, cardiorespiratory fitness, and curl-up performance. Furthermore, only inactive adolescents perceived an increase in their level of physical activity. The measurement protocol was registered prior to the start of the intervention at ClinicalTrials.gov (code: NCT04860128).

## 1 Introduction

Physical inactivity, is a concept that tends to be confused with sedentary lifestyle (1). This term is defined as the failure to meet the minimum guidelines on physical activity practice established by organizations such as the World Health Organization (WHO) or the American College of Sports Medicine (ACSM) (2). Although the scientific literature on the health benefits of physical activity is numerous, physical inactivity has grown exponentially in recent decades in the world population (3, 4). Thus, a high percentage of the world’s population is currently considered inactive, with women accounting for a large part of this percentage (5, 6). As a consequence of the above, physical inactivity is currently the fourth leading cause of death in the world (2). In addition, is also one of the most determining factors in the prevalence of chronic diseases such as obesity, hypertension, diabetes, or metabolic syndrome, becoming one of the major public health problems (5, 7–9). Not surprisingly, it is estimated that given the consequences of physical inactivity on health, this factor generates a health expenditure of around 53.8 million euros/dollars worldwide (10).

This situation is especially relevant during adolescence, as it is a crucial stage for the establishment of physical activity, along with other healthy behaviors, preventing the onset of chronic diseases (7–9, 11). The adolescent population has been greatly affected by physical inactivity in recent years (12). In addition, puberty has become one of the critical stages in which a higher percentage of adolescents drop out of physical activity (13). The COVID-19 pandemic greatly contributed to this, as it drastically affected the period of movement of this population (14). The main consequences of the pandemic were the decrease in physical activity and the increase of screen time, resulting in an increase in physical inactivity and sedentary behaviors in adolescents (13, 15, 16). Although the confinement eventually ended, some of the unhealthy habits acquired by adolescents during the pandemic seem to persist (17). Thus, the pre-pandemic levels of physical activity have not been recovered in this population (18).

As a consequence of the above, 80% of adolescents currently do not meet the minimum physical activity recommendations established by the WHO (6). These recommendations include 60 min of moderate to vigorous intensity physical activity per day to promote physical fitness, a healthy anthropometry and body composition, and a greater adherence to the Mediterranean diet (AMD) (6, 19, 20). For children and adolescents, these recommendations can be undertaken as part of leisure and recreational activities, physical education, transport (cycling, walking and hiking) or household chores (21). Regarding transport, previous research has shown that walking between 11,500 and 14,000 steps per day is the optimal measure that corresponds to the recommendations for moderate to vigorous intensity physical activity (22). In addition, walking becomes relevant in this population because has shown that programs of 8 weeks or more can improve cardiorespiratory fitness, adherence to diet pattern and body mass index (BMI) in adolescents (21, 23, 24). This is because walking has been shown to increase physiological activity and the process of energy expenditure, with potential long-term weight control (25, 26).

Even with the wide variety of possibilities, the minimum levels of physical activity are not reached in the adolescent population, which is even more worrying for females and older adolescents, as this inactivity could affect their health status (27, 28). In this context, the subject of physical education during the compulsory education stage can contribute toward the acquisition of healthy lifestyle habits and the promotion of physical activity in the adolescent population (29–31). However, in Spain the educational curriculum only considers 2 h of physical education per week, which is insufficient to increase the time of motor engagement and comply with WHO recommendations (32, 33). For this reason, previous research has promoted the practice of school and out-of-school physical activity outside physical education hours. In this regard, active recess, physical activity programs in out-of-school leisure time, and active travel to school programs stand out (34–36). All of them have been shown to be effective strategies to increase the daily time dedicated to moderate to vigorous intensity physical activities in the adolescent population, allowing for a reduction in the rate of obesity (37, 38).

In recent years, the inclusion of mobile step-tracking apps has become a new trend in the interventions used to promote the practice of physical activity among adolescents (39, 40). These studies are characterized by two aspects: (a) the use of step tracker app through mobile phones in the classroom (41, 42); and (b) the use of these step tracker apps outside school hours (43). Regarding the former, the results have not been very encouraging, as they have shown the disadvantage that the step tracker mobile apps used are not designed for use in an educational center, limiting the possible benefits (44). Regarding the second aspect, it has shown significant benefits on the level of physical activity, physical fitness, kinanthropometry and body composition of adolescents, mainly when its use is promoted by the physical education subject (45, 46).

The usefulness of mobile apps for the promotion of physical activity in the adolescent population lies in the following reasons: (a) mobile phones are fully integrated in the lives of adolescents (47) and apps have proven to be useful for the generation of a healthy habit in which the mobile phone is a fundamental part (46); (b) one of the main reasons for the abandonment of physical activity programs is the lack of motivation of the participants (48), as many of them do not perceive themselves as sufficiently competent or autonomous during sport activities (49), so apps can solve this situation by enabling adolescents to practice autonomously and in activities of their own choice (50); (c) the possibility of interacting with other users through the app is another reason for using it (50), as the relationship with others is one of the main reasons for this population to practice physical activity (51); and (d) the use of the app promoted by the physical education subject in schools allows adolescents who take the intervention to obtain an academic reward, which provides greater motivation to continue using it due to the increase in external regulation (52), avoiding the initial abandonment of the intervention. In this way, mobile apps for the promotion of physical activity in adolescents could fulfill the key aspects of the theory of self-determination (53) by developing in adolescents a feeling of competent, autonomous and social relations, which could encourage adolescents to make more consistent use of these apps than other types of interventions that do not promote these three issues.

Despite the benefits found with the use of mobile applications promoted from the subject of physical education, this area of research has been under explored, and there are still many unknowns to be resolved (40, 45). Little is known about whether the previous level of physical activity of adolescents could be a determining factor in the use of step tracker mobile apps. This is because previous studies have shown differences in the effectiveness of physical activity intervention depending on the previous physical activity level of the adolescents (54). In this respect, a lower level of physical activity at the beginning of the intervention program, commonly measured by questionnaires recording physical activity in the last week, is related to higher effectiveness of intervention in adolescents (54–56). Only one previous study used step tracker mobile apps with inactive adolescents between 12 and 15 years of age, obtaining improvements in their cardiorespiratory fitness and decreasing their BMI (57). However, this research did not include a group of initially active adolescents to compare whether the benefits were truly superior in the initially inactive group.

Furthermore, although it has been found that the benefit of using this type of mobile application in physical education may be higher among females, and that it may depend on the number of steps taken, no studies have analyzed whether these factors may also differentiate between active and inactive students (58). In addition, one aspect that has not been considered in research conducted with mobile applications in adolescents is the effect that the maturational process may have on the changes observed. Thus, the duration of the interventions with mobile apps is not excessively long, varying between eight and 12 weeks, which leads to consider that the maturational process plays a fundamental role in the changes found in the adolescents, but there is no scientific evidence to contrast this (59, 60). On the other hand, it has been observed that the use of different mobile applications can lead to small changes in the different study variables. However, it is not known whether this could be affected by the level of physical activity of the adolescents who use them (61). Therefore, the objectives of the present research were: (a) to analyze the differences in the level of physical activity, AMD, anthropometry, body composition, and physical fitness of active and inactive adolescents achieved by a 10-week program of after-school use of step tracker mobile apps promoted from the physical education subject; and (b) to determine the influence of gender, maturity, the app used and the number of steps taken with the mobile apps on the study variables.

According to the aims of the present research, it is expected that the use of mobile step-tracking apps will be more effective on the study variables in inactive adolescents, since their starting level of physical activity is lower than that of active ones (H1); and that gender, maturity, app used and the number of steps taken with the mobile apps will influence the changes produced in the study variables after the use of the mobile apps (H2).

## 2 Materials and methods

### 2.1 Design

A randomized controlled trial (RCT) was conducted, consisting of a 10-week intervention in which step tracker mobile apps were used to promote physical activity in the adolescent population. An experimental group (EG) and a control group (CG) were used in the research. With the pre-test measurement of physical activity level, the EG and CG adolescents were divided into active and inactive according to the cut-off values of the “Physical Activity Questionnaire for Adolescents” (PAQ-A) (55). Participants were measured before starting the intervention (pre-test) and after finishing the intervention (post-test). In each measurement, the level of physical activity, kinanthropometric and body composition variables, AMD, and physical fitness were assessed. EG active and inactive participants used one of the step tracker mobile apps selected for the research (Strava, Pacer, MapMyWalk, and PokémonGo). The adolescents used the apps for 10 weeks, a minimum of three times per week, covering the proposed target distance for each week, which was incremental as the intervention progressed. The CG active and inactive adolescents did not use any step tracker mobile apps and continued to attend physical education classes as usual.

The study design followed the principles of the Declaration of Helsinki and was previously approved by the institutional committee of the Catholic University of Murcia (code: CE022102). In addition, the measurement protocol was registered prior to the start of the intervention at ClinicalTrials.gov (code: NCT04860128), and complied with the Consolidated Standards of Reporting Trials (CONSORT) guidelines (62).

The sampling was non-probabilistic by convenience. The intervention took place in two public centers of compulsory secondary education in the Region of Murcia. These centers had a high number of students enrolled in compulsory secondary education (more than 200 students per center). For their selection, the compulsory secondary school in the Region of Murcia with the largest sample of adolescents was contacted and, if the selected school did not wish to participate, the next public school with the largest number of students in compulsory secondary education would be chosen. However, the two centers initially contacted agreed to participate in the research. Once both centers were selected, a meeting was held with each management team to inform them of the research objectives and procedures. Once approval was received, a meeting was held with the teachers in charge of physical education and, subsequently, with the students and their parents. Participation was completely voluntary and the adolescents who were interested provided informed consent signed by themselves and their parents authorizing data collection and subsequent publication of the anonymous data.

### 2.2 Participants

To calculate the sample size, we used the methodology from previous studies based on the standard deviation (SD) (63). Thus, the SD of previous studies in which physical activity practice was promoted by means of step tracker mobile apps (SD = 0.66) was used (64). For an estimated error (d) of 0.06 and a confidence interval of 95% (95% CI), the minimum sample needed for conducting the study was 380 adolescents.

**Figure 1:**
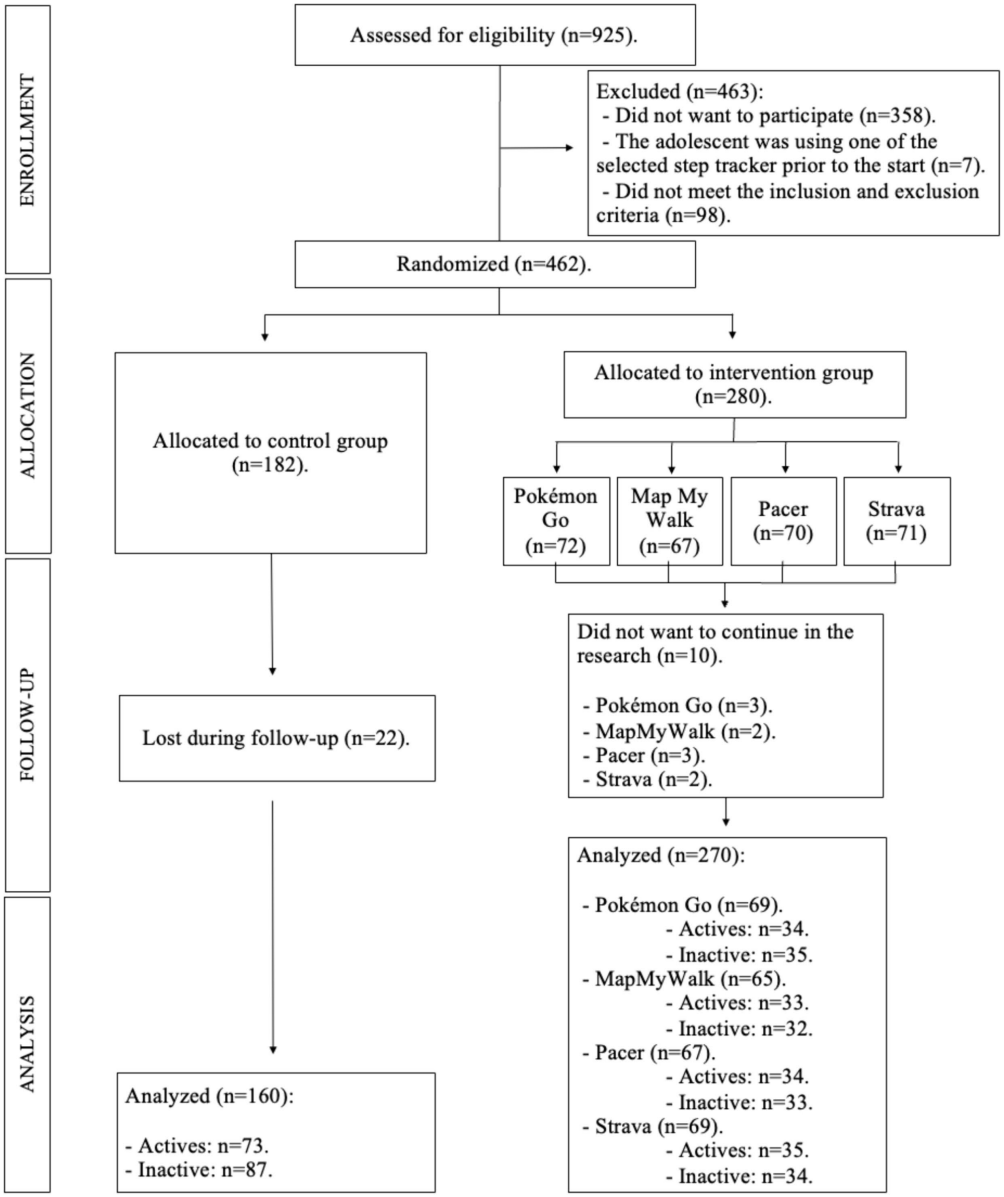
Flow diagram of the sample selection.

A total of 925 adolescents were potentially eligible between the two schools. The initial participation was 462 adolescents, with 430 adolescents participating in the research at the end (270 EG; 160 CG). The number of adolescents who were active and inactive at the beginning of the study was similar (209 active and 221 inactive). The participation of males and females was homogeneous (225 males and 205 females). The age of the adolescents was between 12 and 16 years (mean age: 13.76 ± 1.41 years). The sample selection flow diagram is shown in Figure 1.

The inclusion criteria were (a) age between 12 and 16 years old; (b) not presenting any disease or pathology that would hinder participation; and (c) attending compulsory secondary education. The exclusion criteria were (a) not regularly attending physical education classes during the school year (compulsory for more than 80% of the sessions); (b) not having a cell phone, among the adolescents in the EG; (c) changing educational center; (d) starting some type of systematic physical activity, understood as an activity performed more than three times a week for a duration of more than 60 min a day (such as a specific sport or going to the gym), but without considering the fact of starting to walk, since this was one of the objectives of the research; (e) abandoning the physical activities that were performed regularly before the beginning of the research; and (f) using one of the step tracker mobile apps designated for the research prior to the start of the research. Starting or abadoning any type of systematic physical activity was asked to adolescents in the post-test, together with the PAQ-A questionnaire. A specific question was included after the PAQ-A specifying what was meant by systematic physical activity, which adolescents were asked to answer dichotomously (yes or no).

### 2.3 Randomization and blinding

The randomization process was carried out by the principal investigator in the presence of other investigators not involved in the study, using a computer-generated random number table. For this purpose, the adolescents from each of the schools were randomized into EG and CG, so that in both schools there were adolescents from both groups, thus avoiding that the teaching plans proposed in each school in the physical education subject could contaminate the data. In addition, randomization was done by class, so that all adolescents belonging to that class became part of the CG or one of the EG groups. A second randomization was carried out in the EG to establish the step tracker mobile apps to be used by each of the classes (Strava, Pacer, MapMyWalk, PokémonGo). Four classes were randomly assigned to each application, achieving a homogeneous number of adolescents per step tracker mobile apps at the beginning of the intervention (Figure 1).

Pre-test measurements were performed after randomization. The researchers involved in the measurements were blinded as to which group each adolescent belonged to. They also did not know whether they were active or inactive at baseline, as this was determined after measurements for both EG and CG. In the post-test measurements, the researchers were blinded to whether the adolescents belonged to the EG or CG, to whether they were active or inactive before the start of the investigation, and to the score obtained by each adolescent in the pre-test measurements. The researchers in charge of monitoring the EG intervention were also blinded to the scores obtained by the adolescents in the measurements.

### 2.4 Instruments

The tests and instruments included are justified on the basis that previous research with mobile applications has shown significant changes in these variables (58, 61). However, the present study aims to determine whether there are differences in the effectiveness of the intervention in active and inactive adolescents. In addition, the inclusion of covariates in the present study will allow to determine whether gender, maturity status, app used and distance traveled with the app significantly influence the results, or whether the changes are the result of the intervention.

#### 2.4.1 Questionnaire measurements

The “Physical Activity Questionnaire for Adolescents” (PAQ-A) was used to assess the level of physical activity in the adolescent population (65). This questionnaire is composed of 9 items. The arithmetic mean of the first eight items provides the final physical activity score. The ninth item allows to know if the adolescents had had any difficulty in performing physical activity during the week prior to completing the questionnaire. This questionnaire was used to determine active and inactive adolescents. Thus, adolescents were considered to be active when they scored 2.75 or higher on the PAQ-A, while they were considered inactive when they scored below 2.75 (55). This questionnaire was previously validated in an adolescent population and presents an intraclass correlation coefficient of 0.71 for the final score of the questionnaire (66).

AMD was assessed using the Mediterranean Diet Quality Index (KIDMED). This questionnaire is composed of 16 items that are completed with a 1 or 0 depending on whether the statement indicated is true (1) or not (0). Of the 16 items, 12 have a positive connotation (+1), favoring a good AMD, while four have a negative connotation (−1), hindering a good AMD. For this reason, the final score of the questionnaire ranges from 0 to 12 points, with a higher score referring to a better AMD. This questionnaire presents adequate reliability and reproducibility for use in the adolescent population (α = 0.79 and kappa: 0.66) (67).

#### 2.4.2 Kinanthropometric and body composition measurements

Adolescent body composition and anthropometry were measured by anthropometrists accredited by the International Society for the Advancement of Kinanthropometry (ISAK) (levels 3 to 4). The measurement consisted of body mass and height; triceps, thigh and leg skinfolds; and relaxed arm, waist, mid-thigh and leg girths (68). Kinanthropometric measurements were used to calculate the following variables: BMI, muscle mass, fat mass, sum of 3 skinfolds (triceps, thigh, and leg), corrected arm, thigh and calf girths, and waist-to-height ratio (waist girth/height) (69, 70).

All the measurements were performed following the protocol established by ISAK (68). A skinfold caliper (Harpenden, Burgess Hill, United Kingdom) with an accuracy of 0.2 mm was used to measure skinfolds; a Lufkin W606PM inextensible tape (Lufkin Industries) with an accuracy of 0.1 cm was used to measure girths; a TANITA BC 418-MA segmental scale (TANITA, Tokyo, Japan) with an accuracy of 100 g was used to measure body mass; and a SECA 213 stadiometer (SECA, Hamburg) with an accuracy of 0.1 cm was used to measure height. All instruments were calibrated prior to the pre- and post-test measurements. In the pre and post measurements, each anthropometrist measured the same subjects to minimize inter-rater error. Intra- and inter-evaluator technical measurement error (TME) was calculated in a subsample. For the basic measurements, the intra- and inter-evaluator TME was 0.03 and 0.05%, respectively; 1.23 and 1.99% for skinfolds, respectively; and 0.03 and 0.05% for perimeters, respectively.

The maturity offset was calculated according to the procedure established by Mirwald et al. and using gender-specific formulas: −9.37 + 0.0001882 × ((height-sitting height) × sitting height) − 0.0022 × (age × (height − sitting height)) + 0.005841 × (age × sitting height) − 0.002658 × (age × weight) + 0.07693 × (weight/height) (71). The result of the maturity offset equation is expressed in years from the age at peak height velocity (APHV) when the result is positive, and in years to the APHV when the result is negative (71).

#### 2.4.3 Physical fitness test

Five researchers with previous experience in the supervision of physical fitness testing were in charge of the pre and post measurements. To avoid inter-rater bias, each test was assigned to a researcher and all adolescents were familiarized with the test prior to measurement.

The 20-m shuttle run test was used to assess the cardiorespiratory capacity of the participants (72). This is a maximal incremental test with high validity and reliability for use with adolescents (73). The test ends when the subject reaches exhaustion or is unable to complete the 20-m distance two consecutive times before the beep sounds. The last stage at which the subject finishes the test is used to predict maximal oxygen uptake (VO2 max) using the formula by Léger et al. (72).

To measure upper limb strength, two tests were carried out. First, the manual handgrip strength, which is considered a valid and reliable measurement in adolescents (74). The test requires adolescents to apply a maximum manual grip force with the elbow fully extended on a Takei Tkk5401, as this is the optimal position to produce the maximum force (75). All adolescents performed the test with both hands separately. Secondly, the push-up test was carried out. The test had a duration of 1 min. The number of final repetitions in which the elbow was fully extended and flexed during execution was counted (76).

Lower body power was measured using the countermovement jump (CMJ). The test was carried out on a force platform with a sampling frequency of 200 Hz (MuscleLab, Stathelle, Norway). The objective was to reach the highest vertical jump height. According to the guidelines in Barker et al., the hands were to be kept on the hips throughout the flight phase, the knees and ankles were to be fully extended and the adolescents were to keep their backs fully straight (77).

The curl-up test was used to assess the strength-resistance of the abdominal musculature. The adolescents had to perform as many trunk flexions as possible until they reached exhaustion, or until the end of 1 min. Those repetitions in which the upper back was no longer in contact with the ground were considered valid (78).

### 2.5 Measurement procedure

First, the adolescents completed the PAQ-A and the KIDMED. Subsequently, the kinanthropometric assessment was performed. Once finished, the adolescents were provided an explanation corresponding to the physical fitness tests and were familiarized with the execution of the handgrip, push up, CMJ, and curl up tests. Once the familiarization was completed, a progressive warm-up consisting of running and joint mobility involved in the physical fitness tests was performed, and two measurements of each of the tests were taken. The best score obtained was selected as the final value. Between repetitions of the same test, 2 min of rest were allowed, while 5 min were allowed between the different tests. The order in which the tests were conducted was randomized for each adolescent. Once the handgrip, push-ups, CMJ, and curl up tests were completed, a single repetition of the 20-m shuttle run test was performed, as this is a maximal incremental test whose fatigue may influence the performance of the rest of the tests. The recommendations by the National Strength and Conditioning Association (NSCA) were followed to establish the physical fitness assessment protocol, as it is based on the metabolic demands of each test, as well as the fatigue generated by the tests and the time required for recovery (79).

To minimize interference from possible contaminating variables, the pre- and post-test measurements were performed under as similar conditions as possible. Thus, to complete the PAQ-A and KIDMED questionnaires, a classroom in the school was used in which there was no noise, and the researchers only answered the adolescents’ questions, but in no case did they influence their answers. The changing rooms of the sports hall were used for kinanthropometric measurements, and a stable temperature was maintained during all the measurements. The measurements were always taken between 8:30 a.m. and 2:30 p.m. using physical education class hours, so that each class group was measured in the pre- and post-test at the same time, to avoid the time of measurement inducing changes in kinanthropometric and body composition variables (80, 81). For the physical fitness tests, the covered sports pavilion was used to prevent the weather conditions from influencing the performance of the tests.

### 2.6 Mobile application intervention

The initial sample (*n* = 462) was divided into EG (*n* = 280) and CG (*n* = 182). The apps used by the EG adolescents were: Pokémon Go^®^, Pacer^®^, Strava^®^, and MapMyWalk^®^. For the use of the mobile applications, the adolescents carried their mobile device with them while walking the agreed distance for each day. The selection of this device was based on the fact that a high percentage of adolescents have a mobile device, but not all have watches or other wearables that could be used for the same purpose (82, 83).

Four different mobile apps were included to find out if there were significant differences between these when used by active and inactive adolescents. This is because previous research has shown that there may be small differences in the study variables when used in the general adolescent population (61). Four apps that included a high number of behavior change techniques were selected, based on previous studies, and that were available for android and apple, with the aim of finding out if there were differences between “similar” apps (84). In addition, the study design was not compromised as it is a small number of groups and covariates, being small the amount of information that can be lost during the execution (85). Since each school class consisted of a different number of adolescents, there were small sampling differences in EG adolescents at the beginning of the research (Pokémon Go: *n* = 72; MapMyWalk: *n* = 67; Pacer: *n* = 70; Strava: *n* = 71).

Prior to the start of the intervention, the adolescents of the EG were given a brief explanation of the application to be used and were shown how it worked and how to record the distance traveled. The adolescents then began the 10-week intervention in which they used the corresponding step tracker mobile apps after school hours a minimum of three times per week. Ten weeks was chosen because previous research has shown that interventions of short (6–12 weeks) and medium (12–26 weeks) duration are more effective than those of longer duration (more than 26 weeks) (86). Therefore, taking into account the academic year of the adolescents and the possibility to perform the pre- and post-test measurements, a duration of 10 weeks was chosen. The distance to be completed in each session was increased by 600 steps each week, starting with 7,100 steps per day in the first week and ending with 12,500 steps per day in the tenth week. This distance was used in previous research with step tracker mobile apps and was chosen because the target daily distance of the tenth week is the minimum distance established to stop being considered inactive (58, 87). Since the applications recorded the distance in kilometers, the adolescents were given the distance to be covered in kilometers (week 1: 4.57 km per session; week 10: 8.00 km per session), considering that 1 km corresponds to approximately 1,565 steps in this population (88). Regarding step tracker mobile apps, Pokémon Go was selected because it has been shown to be an effective game for increasing the level of physical activity and the number of daily steps in children and adolescents (89, 90); while Pacer, Strava and MapMyWalk were chosen because they include between 8 and 10 techniques for behavior change (84, 89, 90). The CG continued to attend physical education classes as normal and to perform the physical activity they were doing prior to the start of the investigation, but did not use any step tracker mobile apps. After 10 weeks of intervention, physical activity level, anthropometry, body composition and physical fitness of the CG and EG were measured again.

The use of the mobile applications was encouraged in the physical education course, with the adolescents who completed the intervention receiving up to one extra point in their final grade. This is similar to previous research and is due to the fact that rewards have been shown to be a great incentive to motivate adolescents in physical activity interventions (91, 92). Even so, not all EG adolescents completed the required distance each week, but they were not excluded from the research. These adolescents were measured at post-test and continued to be part of the *n* of the research. The intervention completion rate was different between applications. Of the 462 adolescents who initiated the study (Pokémon Go: *n* = 72; Map My Walk: *n* = 67; Pacer: *n* = 70; Strava: *n* = 71; CG: *n* = 182), 430 completed the investigation (6.93% attrition). Of the adolescents who completed the research, 160 belonged to the control group (12.09% attrition), 69 to Pokémon Go (4.17% attrition), 65 to Map My Walk (2.99% attrition), 67 to Pacer (4.29% attrition) and 69 to Strava (2.82% attrition).

### 2.7 Statistical analysis

The normality of the variables was analyzed using the Kolmogorov–Smirnov test, and kurtosis and skewness analysis. As the data followed a normal distribution, parametric tests were used for analysis. Mean and standard deviation (M ± SD) were used as descriptive values for the sample. To analyze the differences in distance traveled depending on whether the adolescents were active or inactive, as well as on the mobile application used, a Student’s *t*-test for independent samples and an ANOVA were used, respectively. Two Student’s *t*-test for independent samples were performed to determine baseline differences in all the study variables between active and inactive adolescents, as well as between adolescents belonging to the EG and CG. A MANOVA was performed to analyze differences in the study variables between adolescents who were active and inactive and who belonged to the CG or EG. Subsequently, a MANCOVA was carried out including four covariates: gender (male or female), maturity (in years from the APHV), app used (Strava, Pacer, MapMyWalk, and Pokémon Go) and distance covered with the use of the app (based on steps taken during the study). An ANCOVA was performed to analyze whether the change observed in the EG between the pre- and post-test was significantly different from that observed in the CG, both in the active and inactive groups, including the covariate gender and maturity. And to analyze whether there were significant differences in the study variables between the different apps selected, firstly, a MANOVA was performed, taking into account the level of physical activity and the app used; and secondly, an ANOVA was performed to establish whether the change between pre-post in each app was significantly different from the change in the others. Partial Eta squared (η^2^) was used to determine the effect size, and was defined as small: ES ≥ 0.10; moderate: ES ≥ 0.30; large: ≥ 1.2; or very large: ES ≥ 2.0, with an error of *p* < 0.05 (93). Statistical significance was established at *p* < 0.05. The statistical analysis was performed using the SPSS statistical package (v. 25.0; SPSS Inc., IL).

## 3 Results

The average distance traveled by the adolescents during the intervention was 146.73 ± 17.96 km. In the EG of active adolescents, the average distance traveled was 147.52 ± 19.24 km, while in the EG of inactive adolescents, the average distance traveled was 145.23 ± 16.78 km. Based on the application used, the average distance traveled by users was 146.91 ± 14.35 km on Pokémon Go, 148.27 ± 16.83 km on Strava, 148.64 ± 19.42 km on Pacer and 144.53 ± 17.65 km on MapMyWalk. There were no significant differences in distance traveled between the EG groups (*p* = 0.864), nor between the different applications used (*p* = 0.657).

At baseline, significant differences were found between active and inactive adolescents in physical activity level (*p* < 0.001), AMD (*p* = 0.004), corrected arm girth (*p* = 0.023), corrected thigh girth (*p* = 0.002), corrected calf girth (*p* = 0. 003), fat mass (*p* < 0.001), sum of 3 skinfolds (*p* < 0.001), muscle mass (*p* < 0.001), VO2 max (*p* < 0.001), handgrip (right: *p* = 0.003; left: *p* < 0.001), CMJ (*p* = 0.001), curl up (*p* = 0.003) and push up (*p* < 0.001). No differences were found in body mass (*p* = 0.849), height (*p* = 0.118), waist-height ratio (*p* = 0.964) or BMI (*p* = 0.430).

By randomly dividing the active and inactive sample between EG and CG, the comparison between EG and CG at baseline showed no differences in physical activity level (*p* = 0.209), body mass (*p* = 0.063), height (*p* = 0.058), BMI (*p* = 0.071), waist/height (*p* = 0.997), corrected arm girth (*p* = 0.512), corrected thigh girth (*p* = 0.946), corrected calf girth (*p* = 0.297), fat mass (*p* = 0.083), sum of 3 skinfolds (*p* = 0.065), muscle mass (*p* = 0.781), V02 max. (*p* = 0.144), handgrip (right: *p* = 0.902; left: *p* = 0.800), CMJ (*p* = 0.950), curl up (*p* = 0.893) nor push up (*p* = 0.339), but differences in AMD were found (*p* < 0.001), with EG adolescents showing higher AMD before the start of the research.

### 3.1 Intra-group differences for active and inactive adolescents

Table 1 shows the differences between the pre- and post-test in the EG and CG of active and inactive adolescents. Thus, the EG of inactive adolescents showed a significant increase in the level of physical activity, as well as an increase in body mass, height, BMI, corrected arm girth, corrected thigh girth, muscle mass, Vo2 max, handgrip right and left hand, curl up and push up; while significantly decreasing the sum of 3 skinfolds after the intervention. As for the CG of inactive adolescents, a significant increase in physical activity level, body mass, BMI, corrected arm girth, corrected leg girth, muscle mass, Vo2 max and push-up was found after the 10-week period.

**Table 1:** Pre and post-test differences in the physical activity level, adherence to Mediterranean diet, body composition and kinanthropometric variables, and physical fitness in active and inactive adolescents according to the group (intra-group differences).

Variable	Group	Pre	Post	Pre-post diff.	95% CI diff.	η^2^	*p*-value
Physical activity	EG-Inactive	2.19 ± 0.48	2.50 ± 0.52	−0.31 ± 0.05	−0.400; −0.221	0.105	<0.001
	CG-Inactive	2.17 ± 0.47	2.36 ± 0.71	−0.19 ± 0.06	−0.306; −0.070	0.024	0.002
	EG - Active	3.23 ± 0.35	3.21 ± 0.44	0.02 ± 0.05	−0.083; 0.126	0.001	0.688
	CG - Active	3.23 ± 0.39	3.03 ± 0.62	0.21 ± 0.06	0.091; 0.321	0.030	<0.001
AMD	EG-Inactive	6.49 ± 2.48	6.52 ± 2.40	−0.03 ± 0.22	−0.461; 0.402	0.001	0.894
	CG-Inactive	5.41 ± 2.44	5.39 ± 2.82	0.01 ± 0.29	−0.556; 0.581	0.001	0.965
	EG-Active	7.34 ± 2.31	7.25 ± 2.72	0.09 ± 0.26	−0.413; 0.592	0.001	0.728
	CG-Active	6.20 ± 2.59	6.10 ± 2.84	0.11 ± 0.28	−0.446; 0.663	0.001	0.701
Body mass	EG-Inactive	55.65 ± 13.23	56.60 ± 13.01	−0.95 ± 0.16	−1.270; −0.636	0.083	<0.001
	CG-Inactive	51.51 ± 9.29	52.14 ± 9.18	−0.63 ± 0.21	−1.046; −0.212	0.022	0.003
	EG-Active	55.23 ± 12.28	56.10 ± 12.18	−0.87 ± 0.19	−1.238; −0.501	0.053	<0.001
	CG-Active	53.42 ± 11.82	54.63 ± 11.71	−1.22 ± 0.21	−1.632; −0.803	0.079	<0.001
Height (cm)	EG-Inactive	162.23 ± 9.32	162.89 ± 9.14	−0.66 ± 0.14	−0.940; −0.389	0.055	<0.001
	CG-Inactive	160.07 ± 8.68	160.42 ± 8.77	−0.36 ± 0.19	−0.720; 0.006	0.010	0.054
	EG-Active	163.83 ± 8.79	164.67 ± 8.83	−0.84 ± 0.16	−1.159; −0.517	0.064	<0.001
	CG-Active	161.67 ± 8.81	162.60 ± 8.55	−0.38 ± 0.18	−1.289; −0.568	0.062	<0.001
BMI	EG-Inactive	21.07 ± 4.10	21.24 ± 3.96	−0.17 ± 0.06	−0.288; −0.043	0.018	0.008
	CG-Inactive	20.09 ± 3.24	20.29 ± 3.23	−0.20 ± 0.08	−0.365; −0.044	0.016	0.013
	EG-Active	20.55 ± 3.43	20.62 ± 3.30	−0.07 ± 0.07	−0.211; 0.074	0.002	0.344
	CG-Active	20.30 ± 3.43	20.57 ± 3.22	−0.27 ± 0.08	−0.426; −0.107	0.027	0.001
Waist-height	EG-Inactive	0.42 ± 0.05	0.42 ± 0.05	0.00 ± 0.00	−0.001; 0.004	0.004	0.193
	CG-Inactive	0.42 ± 0.04	0.42 ± 0.04	−0.00 ± 0.00	−0.005; 0.001	0.005	0.185
	EG-Active	0.42 ± 0.04	0.42 ± 0.04	0.00 ± 0.00	−0.001; 0.005	0.006	0.129
	CG-Active	0.42 ± 0.04	0.42 ± 0.04	0.00 ± 0.00	−0.001; 0.005	0.005	0.187
Corrected arm girth	EG-Inactive	20.69 ± 2.92	21.02 ± 2.66	−0.33 ± 0.08	−0.496; −0.168	0.040	<0.001
	CG-Inactive	20.30 ± 2.24	20.65 ± 2.21	−0.35 ± 0.11	−0.566; −0.127	0.024	0.002
	EG-Active	21.17 ± 2.61	21.69 ± 2.70	−0.52 ± 0.10	−0.704; −0.326	0.069	<0.001
	CG-Active	21.14 ± 3.01	21.56 ± 2.90	−0.42 ± 0.11	−0.629; −0.202	0.037	<0.001
Corrected thigh girth	EG-Inactive	38.71 ± 4.74	39.69 ± 4.63	−0.98 ± 0.20	−1.361; −0.593	0.061	<0.001
	CG-Inactive	38.24 ± 3.80	38.66 ± 3.54	−0.42 ± 0.26	−0.931; 0.095	0.007	0.110
	EG-Active	39.83 ± 4.54	40.73 ± 4.42	−0.90 ± 0.23	−1.341; −0.457	0.040	<0.001
	CG-Active	40.17 ± 5.96	40.65 ± 4.47	−0.48 ± 0.25	−0.982; 0.018	0.009	0.059
Corrected calf girth	EG-Inactive	28.65 ± 3.90	28.86 ± 2.82	−0.22 ± 0.16	−0.536; 0.102	0.005	0.182
	CG-Inactive	28.03 ± 2.23	28.56 ± 2.14	−0.53 ± 0.22	−0.958 -0.106;	0.015	0.015
	EG-Active	29.36 ± 2.73	29.93 ± 2.78	−0.57 ± 0.19	−0.935; −0.201	0.023	0.003
	CG-Active	29.31 ± 2.95	29.77 ± 2.88	−0.46 ± 0.21	−0.879; −0.048	0.012	0.029
Fat mass (%)	EG-Inactive	24.62 ± 10.84	24.12 ± 10.18	0.50 ± 0.29	−0.077; 1.076	0.007	0.089
	CG-Inactive	21.39 ± 9.21	21.40 ± 8.93	−0.01 ± 0.39	−0.780; 0.762	0.001	0.982
	EG-Active	20.32 ± 9.35	19.58 ± 8.66	0.75 ± 0.34	0.083; 1.411	0.013	0.028
	CG-Active	18.84 ± 10.51	18.26 ± 10.29	0.58 ± 0.38	−0.171; 1.331	0.006	0.130
Sum of 3 skinfolds	EG-Inactive	56.80 ± 28.42	54.96 ± 25.81	1.84 ± 0.78	0.314; 3.362	0.014	0.018
	CG-Inactive	47.79 ± 22.20	48.46 ± 21.87	−0.67 ± 1.04	−2.709; 1.365	0.001	0.517
	EG-Active	45.82 ± 23.60	43.94 ± 20.78	1.88 ± 0.89	0.121; 3.632	0.011	0.036
	CG-Active	42.10 ± 24.88	40.39 ± 23.19	1.72 ± 1.01	−0.267; 3.703	0.007	0.090
Muscle mass (kg)	EG-Inactive	17.40 ± 5.07	18.02 ± 4.96	−0.62 ± 0.11	−0.842; −0.393	0.071	<0.001
	CG-Inactive	16.94 ± 3.78	17.38 ± 3.74	−0.44 ± 0.15	−0.736; −0.136	0.021	0.005
	EG-Active	18.87 ± 4.79	19.67 ± 4.99	−0.81 ± 0.13	−1.066; −0.547	0.089	<0.001
	CG-Active	19.48 ± 5.06	19.99 ± 4.62	−0.51 ± 0.15	−0.802; −0.213	0.029	0.001
Vo2 Max	EG-Inactive	36.54 ± 4.29	37.50 ± 5.24	−0.95 ± 0.27	−1.476; −0.426	0.036	<0.001
	CG-Inactive	36.28 ± 3.94	37.05 ± 4.40	−0.77 ± 0.35	−1.458; −0.073	0.014	0.030
	EG-Active	40.02 ± 4.91	41.20 ± 5.63	−1.18 ± 0.31	−1.783; −0.577	0.041	<0.001
	CG-Active	40.93 ± 4.96	41.53 ± 4.82	−0.60 ± 0.36	−1.307; 0.099	0.008	0.092
Handgrip right hand	EG-Inactive	23.60 ± 7.80	24.92 ± 8.35	−1.33 ± 0.39	−2.092; −0.562	0.029	0.001
	CG-Inactive	23.13 ± 6.63	23.99 ± 6.90	−0.86 ± 0.51	−1.863; 0.151	0.007	0.096
	EG-Active	25.69 ± 7.26	27.38 ± 7.99	−1.69 ± 0.45	−2.580; −0.798	0.034	<0.001
	CG-Active	25.59 ± 7.62	26.48 ± 9.65	−0.89 ± 0.50	−1.877; 0.089	0.008	0.074
Handgrip left hand	EG-Inactive	21.94 ± 7.29	22.84 ± 7.10	−0.89 ± 0.30	−1.490; −0.292	0.021	0.004
	CG-Inactive	22.03 ± 6.11	22.40 ± 7.03	−0.37 ± 0.40	−1.159; 0.419	0.002	0.358
	EG-Active	24.43 ± 6.63	25.18 ± 7.64	−0.75 ± 0.36	−1.444; −0.049	0.011	0.036
	CG-Active	24.27 ± 7.15	25.26 ± 8.51	−0.99 ± 0.39	−1.760; −0.221	0.016	0.012
CMJ	EG-Inactive	20.89 ± 7.45	21.36 ± 8.60	−0.47 ± 0.68	−1.807; 0.869	0.001	0.491
	CG-Inactive	20.97 ± 6.85	21.88 ± 7.71	−0.91 ± 0.90	−2.674; 0.851	0.003	0.310
	EG-Active	23.50 ± 6.96	25.84 ± 6.22	−2.34 ± 0.79	−3.897; −0.780	0.022	0.003
	CG-Active	23.06 ± 8.08	23.14 ± 10.81	−0.08 ± 0.87	−1.802; 1.636	0.001	0.924
Curl up	EG-Inactive	19.25 ± 11.34	23.24 ± 9.79	−3.99 ± 0.93	−5.820; −2.165	0.045	<0.001
	CG-Inactive	18.00 ± 9.82	19.57 ± 10.52	−1.57 ± 1.21	−3.950; 0.811	0.004	0.196
	EG-Active	22.01 ± 11.55	25.91 ± 11.62	−3.90 ± 1.07	−6.006; −1.796	0.033	<0.001
	CG-Active	22.66 ± 12.25	24.32 ± 12.83	−1.66 ± 1.21	−4.039; 0.722	0.005	0.172
Push up	EG-Inactive	4.98 ± 7.62	6.43 ± 8.76	−1.45 ± 0.55	−2.534; −0.360	0.018	0.009
	CG-Inactive	5.40 ± 7.01	7.47 ± 8.19	−2.07 ± 0.72	−3.488; −0.642	0.021	0.005
	EG-Active	8.86 ± 10.85	10.89 ± 11.82	−2.03 ± 0.63	−3.272; −0.787	0.026	0.001
	CG-Active	10.27 ± 10.68	10.72 ± 11.11	−0.45 ± 0.73	−1.895; 0.989	0.001	0.537

BMI, body mass index; Sum of 3 skinfolds, summation of 3 skinfolds; Vo2 max, maximal oxygen consumption; CMJ, countermovement jump.

In the EG of active adolescents, a significant increase in body mass, height, corrected arm girth, corrected thigh girth, corrected leg girth, muscle mass, Vo2 max, handgrip right and left hand, CMJ, curl-up and push up was found; but the percentage of fat mass and the sum of 3 skinfolds decreased significantly after the training. In the CG of active adolescents there was a significant increase in body mass, height, BMI, corrected arm girth, corrected leg girth, muscle mass and handgrip left hand; however, the level of physical activity decreased significantly after the 10-week period.

### 3.2 Influence of the covariates in the intra-group differences for active and inactive adolescents

Table 2 shows the influence of the covariates on the differences between pre and post of the different groups. The inclusion of the covariate gender showed significant influence on the EG evolution of inactive adolescents in the variables of physical activity, body mass, height, BMI, corrected arm girth, corrected thigh girth, muscle mass, sum of 3 skinfolds and all physical fitness variables, except CMJ. In the CG of inactive adolescents, the influence of this covariate was found in the evolution of level of physical activity, body mass, height, BMI, corrected arm girth, corrected leg girth, muscle mass, VO2 max and push-ups. In the EG of active adolescents, influence was observed in the evolution of body mass, height, corrected arm girth, corrected thigh girth, corrected leg girth, fat mass, the sum of 3 skinfolds, muscle mass and all the physical fitness variables. And in the CG of active adolescents, this covariate caused significant changes in the evolution of physical activity level, body mass, height, BMI, corrected arm girth, corrected thigh girth, corrected leg girth, muscle mass and handgrip. This covariate showed no influence on AMD in any of the groups analyzed.

**Table 2:** Influence of the covariates gender, maturity, app used and distance covered with the app on the intra-group differences for active and inactive adolescents.

Variable	Group	Gender			Maturity			App used			Distance covered with the app		
		95% CI diff.	η^2^	*p*-value	95% CI diff.	η^2^	*p*-value	95% CI diff.	η^2^	*p*-value	95% CI diff.	η^2^	*p*-value
Physical activity	EG-Inactive	−0.410; −0.227	0.105	<0.001	−0.410; −0.225	0.035	0.341	−0.397; −0.187	0.070	<0.001	−0.406; −0.221	0.101	<0.001
	CG-Inactive	−0.308; −0.071	0.025	0.002	−0.301; −0.062	0.022	0.538	–	–	–	–	–	–
	EG - Active	−0.081; 0.128	0.001	0.658	−0.086; 0.125	0.000	0.714	−0.080; 0.170	0.001	0.484	−0.088; 0.125	0.001	0.729
	CG - Active	0.099; 0.337	0.032	<0.001	0.091; 0.328	0.030	0.402	–	–	–	–	–	–
AMD	EG-Inactive	−0.514; 0.371	0.001	0.752	−0.482; 0.402	0.000	0.857	−0.584; 0.429	0.000	0.765	−0.470; 0.421	0.001	0.914
	CG-Inactive	−0.563; 0.574	0.001	0.985	−0.492; 0.653	0.000	0.782	–	–	–	–	–	–
	EG - Active	−0.403; 0.604	0.001	0.695	−0.426; 0.583	0.000	0.759	−0.573; 0.631	0.000	0.924	−0.419; 0.606	0.001	0.721
	CG - Active	−0.403; 0.744	0.001	0.559	−0.381; 0.755	0.001	0.518	–	–	–	–	–	–
Body mass	EG-Inactive	−1.336; −0.687	0.088	<0.001	−1.394; −0.756	0.102	<0.001	−1.273; −0.533	0.056	<0.001	−1.311; −0.658	0.083	<0.001
	CG-Inactive	−1.059; −0.226	0.023	0.003	−0.983; −0.159	0.019	0.007	–	–	–	–	–	–
	EG-Active	−1.222; −0.485	0.051	<0.001	−1.238; −0.513	0.055	<0.001	−1.246; −0.368	0.033	<0.001	−1.274; −0.523	0.054	<0.001
	CG-Active	−1.553; −0.694	0.064	<0.001	−1.476; −0.641	0.060	<0.001	–	–	–	–	–	–
Height (cm)	EG-Inactive	−1.050; −0.491	0.071	<0.001	−1.086; −0.539	0.081	<0.001	−0.927; −0.283	0.010	0.076	−0.969; −0.400	0.055	<0.001
	CG-Inactive	−0.741; −0.024	0.011	0.037	−0.639; 0.066	0.007	0.111	–	–	–	–	–	–
	EG-Active	−1.126; −0.492	0.061	<0.001	−1.156; −0.535	0.069	<0.001	−1.145; −0.381	0.007	0.105	−1.184; −0.530	0.064	<0.001
	CG-Active	−1.128; −0.388	0.040	<0.001	−1.093; −0.378	0.041	<0.001	–	–	–	–	–	–
BMI	EG-Inactive	−0.292; −0.041	0.017	0.009	−0.317; −0.068	0.023	0.003	−0.313; −0.027	0.010	0.054	−0.290; −0.037	0.016	0.011
	CG-Inactive	−0.366; −0.043	0.016	0.013	−0.352; −0.031	0.014	0.020	–	–	–	–	–	–
	EG-Active	−0.211; 0.074	0.002	0.348	−0.211; 0.072	0.002	0.332	−0.244; 0.095	0.002	0.391	−0.212; 0.078	0.002	0.367
	CG-Active	−0.431; −0.098	0.025	0.002	−0.394; −0.068	0.020	0.006	–	–	–	–	–	–
Waist-height	EG-Inactive	−0.001; 0.004	0.006	0.142	−0.001; 0.004	0.005	0.183	0.000; 0.005	0.007	0.097	−0.001; 0.004	0.003	0.321
	CG-Inactive	−0.005; 0.001	0.004	0.200	−0.005; 0.001	0.005	0.180	–	–	–	–	–	–
	EG-Active	−0.001; 0.005	0.006	0.141	−0.001; 0.005	0.006	0.129	0.000; 0.006	0.009	0.064	−0.001; 0.004	0.004	0.203
	CG-Active	−0.001; 0.005	0.003	0.309	−0.001; 0.005	0.004	0.221	–	–	–	–	–	–
Corrected arm girth	EG-Inactive	−0.489; −0.152	0.035	<0.001	−0.510; −0.174	0.040	<0.001	−0.538; −0.157	0.012	0.065	−0.494; −0.156	0.036	<0.001
	CG-Inactive	−0.563; −0.124	0.024	0.002	−0.563; −0.122	0.024	0.002	–	–	–	–	–	–
	EG-Active	−0.707; −0.329	0.070	<0.001	−0.702; −0.321	0.068	<0.001	−0.759; −0.309	0.004	0.101	−0.701; 0.316	0.066	<0.001
	CG-Active	−0.656; −0.212	0.037	<0.001	−0.629; −0.188	0.034	<0.001	–	–	–	–	–	–
Corrected thigh girth	EG-Inactive	−1.322; −0.535	0.053	<0.001	−1.345; −0.559	0.056	<0.001	−1.444; −0.552	0.004	0.137	−1.370; −0.579	0.058	<0.001
	CG-Inactive	−0.918; 0.109	0.006	0.122	−0.943; 0.087	0.007	0.103	–	–	–	–	–	–
	EG-Active	−1.354; −0.469	0.041	<0.001	−1.341; −0.451	0.039	<0.001	−1.452; −0.399	0.005	0.169	−1.348; −0.446	0.038	<0.001
	CG-Active	−1.081; −0.042	0.012	0.034	−1.062; −0.030	0.011	0.038	–	–	–	–	–	–
Corrected calf girth	EG-Inactive	−0.559; 0.095	0.005	0.164	−0.588; 0.065	0.006	0.116	−0.644; 0.096	0.005	0.146	−0.499; 0.157	0.003	0.307
	CG-Inactive	−0.963; −0.109	0.016	0.014	−0.941; −0.086	0.014	0.059	–	–	–	–	–	–
	EG-Active	−0.932; −0.196	0.023	0.003	−0.935; −0.195	0.011	0.073	−1.078; −0.203	0.011	0.054	−0.900; −0.152	0.020	0.006
	CG-Active	−0.871; −0.007	0.010	0.046	−0.840; 0.017	0.009	0.060	–	–	–	–	–	–
Fat mass (%)	EG-Inactive	−0.047; 1.136	0.008	0.071	−0.080; 1.102	0.008	0.090	−0.136; 1.204	0.006	0.118	−0.227; 0.957	0.004	0.226
	CG-Inactive	−0.768; 0.776	0.001	0.993	−0.789; 0.762	0.000	0.973	–	–	–	–	–	–
	EG-Active	0.070; 1.401	0.012	0.030	0.069; 1.409	0.012	0.051	−0.001; 1.582	0.010	0.051	−0.048; 1.300	0.009	0.069
	CG-Active	−0.275; 1.287	0.004	0.203	−0.214; 1.340	0.005	0.155	–	–	–	–	–	–
Sum of 3 skinfolds	EG-Inactive	0.332; 3.459	0.015	0.018	0.361; 3.486	0.015	0.076	0.058; 3.598	0.011	0.053	0.100; 3.239	0.011	0.037
	CG-Inactive	−2.697; 1.386	0.001	0.528	−2.755; 1.342	0.001	0.498	–	–	–	–	–	–
	EG-Active	0.103; 3.622	0.011	0.038	0.104; 3.645	0.008	0.118	−0.228; 3.956	0.008	0.081	−0.063; 3.513	0.009	0.059
	CG-Active	−0.441; 3.688	0.006	0.123	−0.434; 3.670	0.006	0.122	–	–	–	–	–	–
Muscle mass (kg)	EG-Inactive	−0.848; −0.386	0.068	<0.001	−0.846; −0.387	0.068	<0.001	−0.886; −0.364	0.022	0.106	−0.831; −0.368	0.064	<0.001
	CG-Inactive	−0.737; −0.135	0.021	0.005	−0.737; −0.135	0.021	0.005	–	–	–	–	–	–
	EG-Active	−1.067; −0.546	0.088	<0.001	−1.067; −0.546	0.089	<0.001	−1.125; −0.507	0.046	0.067	−1.055; −0.525	0.083	<0.001
	CG-Active	−0.814; −0.201	0.027	0.001	−0.810; −0.206	0.028	0.001	–	–	–	–	–	–
Vo2 Max	EG-Inactive	−1.696; −0.645	0.053	<0.001	−1.516; −0.438	0.036	0.084	−1.419; −0.200	0.020	0.009	−1.404; −0.312	0.027	0.002
	CG-Inactive	−1.508; −0.150	0.017	0.017	−1.413; −0.013	0.012	0.066	–	–	–	–	–	–
	EG-Active	−1.728; −0.546	0.040	<0.001	−1.789; −0.574	0.041	0.057	−1.719; −0.285	0.022	0.006	−1.724; −0.499	0.036	<0.001
	CG-Active	−0.921; 0.512	0.001	0.574	−1.291; 0.157	0.007	0.124	–	–	–	–	–	–
Handgrip right hand	EG-Inactive	−2.303; −0.743	0.036	<0.001	−2.125; −0.554	0.028	<0.001	−1.724; −0.939	0.020	0.103	−2.068; −0.488	0.025	0.002
	CG-Inactive	−1.892; 0.113	0.008	0.082	−1.886; 0.150	0.011	0.044	–	–	–	–	–	–
	EG-Active	−2.523; −0.749	0.032	<0.001	−2.659; −0.866	0.037	<0.001	−2.382; −1.259	0.025	0.062	−2.554; −0.737	0.031	<0.001
	CG-Active	−1.613; 0.407	0.003	0.241	−1.925; 0.094	0.006	0.035	–	–	–	–	–	–
Handgrip left hand	EG-Inactive	−1.595; −0.367	0.024	0.002	−1.490; −0.257	0.019	0.006	−1.891; −0.489	0.017	0.076	−1.527; −0.289	0.021	0.004
	CG-Inactive	−1.174; 0.404	0.002	0.338	−1.134; 0.464	0.002	0.040	–	–	–	–	–	–
	EG-Active	−1.420; −0.024	0.010	0.043	−1.469; −0.062	0.012	0.033	1.954; −0.287	0.021	0.069	−1.473; −0.049	0.011	0.036
	CG-Active	−1.652; −0.062	0.011	0.035	−1.832; −0.248	0.017	0.010	–	–	–	–	–	–
CMJ	EG-Inactive	−2.087; 0.652	0.003	0.303	−1.925; 0.767	0.002	0.398	−1.900; 1.242	0.000	0.681	−1.664; 1.097	0.001	0.687
	CG-Inactive	−2.714; 0.805	0.003	0.287	−2.240; 1.250	0.001	0.578	–	–	–	–	–	–
	EG-Active	−3.829; −0.714	0.020	0.004	−3.678; −0.605	0.005	0.106	−3.031; −0.296	0.013	0.073	−3.760; −0.584	0.018	0.007
	CG-Active	−1.487; 2.059	0.001	0.751	−1.684; 1.776	0.000	0.958	–	–	–	–	–	–
Curl up	EG-Inactive	−5.640; −1.897	0.039	<0.001	−5.053; −1.347	0.019	0.071	−6.166; −1.884	0.034	<0.001	−5.676; −1.879	0.038	<0.001
	CG-Inactive	−3.906; 0.856	0.004	0.209	−4.232; 0.515	0.006	0.125	–	–	–	–	–	–
	EG-Active	−6.065; −1.850	0.034	<0.001	−5.899; −1.718	0.012	0.134	−6.460; −1.423	0.024	0.002	−5.865; −1.557	0.029	0.001
	CG-Active	−4.474; 0.453	0.007	0.109	−5.002; −0.167	0.011	0.086	–	–	–	–	–	–
Push up	EG-Inactive	−2.745; −0.519	0.021	0.004	−2.836; −0.614	0.024	0.002	−2.460; 0.065	0.009	0.063	−2.525; −0.266	0.015	0.016
	CG-Inactive	−3.538; −0.693	0.022	0.004	−3.285; −0.427	0.017	0.011	–	–	–	–	–	–
	EG-Active	−3.227; −0.742	0.025	0.002	−3.348; −0.861	0.029	<0.001	−3.197; −0.241	0.014	0.053	−3.257; −0.715	0.024	0.002
	CG-Active	−1.637; 1.364	0.001	0.858	−1.560; 1.386	0.011	0.007	–	–	–	–	–	–

BMI, body mass index; Sum of 3 skinfolds, summation of 3 skinfolds; Vo2 max, maximal oxygen consumption; CMJ, countermovement jump.

The maturity covariate showed significant influence in EG of inactive adolescents in the evolution of body mass, height, BMI, corrected arm and thigh girths, muscle mass, handgrip right and left hand and push-ups. In the CG inactive, this covariates influenced in the evolution of body mass, BMI, corrected arm girth, muscle mass, handgrip right and left hands and push-ups. In the EG active, the influence of this covariate was observed in the evolution of body mass, height, corrected arm and thigh girths, muscle mass, handgrip right and left hands and push-ups. And in the CG active, the influence was significant in the evolution of body mass, height, BMI, corrected arm and thigh girths, muscle mass, handgrip right and left hands, and push-ups. However, this covariate did not show influence in the evolution of physical activity, AMD, waist-height, corrected calf-girth, fat mass, sum of 3 skinfolds, VO2 max., CMJ nor curl up (Table 2).

The covariate app used showed influence in the EG inactive in the involution of physical activity, body mass, VO2 max. and curl up, while in the EG active the differences were significant in the evolution of body mass, VO2 max. and curl up. And the inclusion of the covariable distance covered with the app showed significant influence on the EG of inactive adolescents in the evolution of the variable physical activity, body mass, height, BMI, corrected arm girth, corrected thigh girth, sum of 3 skinfolds, muscle mass and all the physical fitness test, except CMJ. In the EG of active adolescents, influence was observed on the evolution of body mass, height, corrected arm girth, corrected thigh girth, corrected calf girth, muscle mass and all the physical fitness test. Neither of the two covariates showed any influence on AMD in any of the groups analyzed (Table 2).

### 3.3 Inter-group differences (EG vs. CG) in intra-group differences for active and inactive adolescents

Table 3 shows the differences in the change showed by EG and CG in both the active and inactive adolescent groups. The results showed significant differences in the group of active adolescents in BMI, with the change observed being greater in the CG, with a significant effect of the covariates gender and maturity. In the case of the CMJ test for active group, a greater change was found in EG compared to CG, without influence of the covariates. No significant differences were found in the rest of the variables analyzed. Thus, the difference between the intra-group change in EG and CG was not significant for physical activity level and AMD. Neither was it significant for kinanthropometric variables or body composition (body mass, height, waist-height, corrected girths, fat mass, sum of 3 skinfolds or muscle mass), nor for fitness variables (VO2 max, handgrip left and right hands, curl-up or push-up). The covariates gender and maturity also showed no effect on the variables for which the analysis of change was not statistically significant.

**Table 3:** Inter-group differences (EG vs. CG) in the intra-group differences for active and inactive groups.

Variable	Group	Pre-post EG – pre-post CG				Gender covariate			Maturity covariate		
		Diff.	95%CI diff.	η^2^	*p*-value	95%CI diff.	η^2^	*p*-value	95%CI diff.	η^2^	*p*-value
Physical activity	Inactives	−0.07 ± 0.07	−0.206; 0.072	0.003	0.343	−0.208; 0.071	0.003	0.337	−0.213; 0.069	0.003	0.314
	Actives	−0.11 ± 0.07	−0.256; 0.036	0.007	0.138	−0.262; 0.036	0.007	0.136	−0.265; 0.032	0.007	0.124
AMD	Inactives	0.27 ± 0.34	−0.395; 0.927	0.002	0.429	−0.409; 0.917	0.002	0.452	−0.357; 0.981	0.003	0.360
	Actives	0.04 ± 0.35	−0.655; 0.734	0.001	0.911	−0.700; 0.716	0.001	0.982	−0.610; 0.801	0.000	0.790
Body mass	Inactives	−0.40 ± 0.27	−0.932; 0.131	0.006	0.139	−0.972; 0.090	0.008	0.103	−1.038; 0.030	0.010	0.064
	Actives	0.46 ± 0.28	−0.096; 1.021	0.008	0.104	−0.211; 0.922	0.005	0.218	−0.227; 0.899	0.004	0.242
Height (cm)	Inactives	−0.22 ± 0.21	−0.628; 0.186	0.003	0.286	−0.686; 0.107	0.006	0.152	−0.768; 0.030	0.010	0.069
	Actives	0.08 ± 0.22	−0.347; 0.507	0.001	0.713	−0.526; 0.320	0.001	0.632	−0.522; 0.319	0.001	0.635
BMI	Inactives	0.02 ± 0.11	−0.206; 0.240	0.001	0.882	−0.210; 0.239	0.001	0.899	−0.235; 0.216	0.000	0.936
	Actives	0.26 ± 0.12	0.022; 0.491	0.014	0.032	0.011; 0.489	0.012	0.040	−0.013; 0.463	0.010	0.044
Waist-height	Inactives	0.00 ± 0.00	−0.001; 0.007	0.006	0.172	−0.001; 0.007	0.006	0.151	−0.001; 0.007	0.006	0.169
	Actives	0.00 ± 0.00	−0.004; 0.005	0.000	0.846	−0.004; 0.005	0.000	0.705	−0.004; 0.005	0.000	0.825
Corrected arm girth	Inactives	0.06 ± 0.15	−0.246; 0.356	0.001	0.720	−0.237; 0.367	0.001	0.672	−0.259; 0.350	0.000	0.769
	Actives	−0.05 ± 0.16	−0.368; 0.265	0.001	0.750	−0.346; 0.298	0.001	0.883	−0.385; 0.259	0.000	0.702
Corrected thigh girth	Inactives	−0.59 ± 0.36	−1.292; 0.105	0.008	0.095	−1.272; 0.129	0.008	0.110	−1.231; 0.181	0.006	0.144
	Actives	−0.49 ± 0.37	−1.220; 0.247	0.005	0.193	−1.174; 0.320	0.004	0.262	−1.147; 0.342	0.003	0.289
Corrected calf girth	Inactives	0.40 ± 0.30	−0.186; 0.990	0.005	0.179	−0.204; 0.976	0.005	0.199	−0.250; 0.939	0.004	0.255
	Actives	0.01 ± 0.31	−0.609; 0.626	0.001	0.978	−0.665; 0.594	0.001	0.913	−0.689; 0.565	0.000	0.846
Fat mass (%)	Inactives	0.36 ± 0.53	−0.684; 1.403	0.001	0.499	−0.657; 1.437	0.002	0.464	−0.677; 1.438	0.001	0.480
	Actives	−0.01;0.56	−1.108; 1.085	0.001	0.984	−1.047; 1.187	0.001	0.902	−1.102; 1.130	0.000	0.980
Sum of 3 skinfolds	Inactives	1.999 ± 1.41	−0.780; 4.778	0.006	0.158	−0.739; 4.841	0.006	0.149	−0.696; 4.933	0.007	0.140
	Actives	0.04 ± 1.48	−2.882; 2.958	0.001	0.979	−2.799; 3.152	0.001	0.907	−2.785; 3.154	0.000	0.903
Muscle mass (kg)	Inactives	−0.16 ± 0.20	−0.563; 0.249	0.002	0.447	−0.568; 0.248	0.002	0.441	−0.547; 0.275	0.001	0.516
	Actives	−0.33 ± 0.22	−0.752; 0.102	0.007	0.135	−0.767; 0.103	0.007	0.134	−0.733; 0.135	0.005	0.176
Vo2 Max	Inactives	−0.43 ± 0.45	−1.312; 0.453	0.003	0.339	−1.420; 0.316	0.005	0.212	−1.337; 0.451	0.003	0.331
	Actives	−0.57 ± 0.47	−1.492; 0.362	0.004	0.231	−1.818; 0.035	0.011	0.059	−1.525; 0.362	0.004	0.226
Handgrip right hand	Inactives	−0.39 ± 0.56	−1.491; 0.708	0.001	0.484	−1.544; 0.662	0.002	0.432	−1.407; 0.818	0.001	0.603
	Actives	−0.61 ± 0.59	−1.762; 0.550	0.003	0.303	−1.912; 0.440	0.005	0.219	−1.661; 0.687	0.002	0.415
Handgrip left hand	Inactives	−0.50 ± 0.43	−1.337; 0.341	0.004	0.244	−1.365; 0.319	0.004	0.222	−1.266; 0.430	0.003	0.333
	Actives	0.50 ± 0.45	−0.378; 1.385	0.004	0.262	−0.461; 1.335	0.003	0.339	−0.293; 1.496	0.005	0.187
CMJ	Inactives	0.48 ± 0.69	−0.864; 1.830	0.001	0.481	−0.933; 1.765	0.001	0.545	−0.752; 1.972	0.002	0.379
	Actives	1.57 ± 0.72	0.151; 2.982	0.014	0.030	−0.052; 2.827	0.011	0.059	0.285; 3.159	0.016	0.069
Curl Up	Inactives	−2.48 ± 1.66	−5.735; 0.780	0.007	0.136	−5.676; 0.864	0.006	0.149	−5.145; 1.401	0.004	0.261
	Actives	−1.84 ± 1.74	−5.258; 1.588	0.003	0.292	−5.133; 1.843	0.003	0.354	−4.547; 2.360	0.001	0.534
Push Up	Inactives	0.69 ± 0.95	−1.181; 2.551	0.002	0.471	−1.298; 2.436	0.001	0.549	−1.539; 2.210	0.000	0.725
	Actives	−1.30 ± 1.00	−3.264; 0.658	0.005	0.192	−3.601; 0.381	0.007	0.113	−3.708; 0.248	0.009	0.086

BMI, body mass index; Sum of 3 skinfolds, summation of 3 skinfolds; Vo2 max, maximal oxygen consumption; CMJ, countermovement jump.

### 3.4 Differences in the study variables in the groups of active and inactive adolescents according to the app used

Table 4 shows the pre-post differences for each of the variables according to the different mobile apps used by active and inactive adolescents. There was a significant increase in the level of physical activity in the inactive group with all the apps (*p* < 0.001–0.032). In body mass, there was an increase in Strava (*p* < 0.001), Pacer (*p* = 0.003) and MapMyWalk (*p* = 0.007) inactives, as well as in Pokémon Go (*p* = 0.003) and Strava (*p* < 0.001) actives. Significant increases occurred in all groups in height (*p* < 0.001–0.049), except in the MapMyWalk actives group (*p* = 0.105). In corrected arm girth there was a significant increase in Pacer (*p* = 0.009) and MapMyWalk (*p* = 0.011) inactives, as well as in Pokémon Go (*p* < 0.001), Strava (*p* = 0.006) and MapMyWalk (*p* = 0.004) actives. In the corrected thigh girth the significant increase occurred in the group of Strava (*p* = 0.004) and Pacer (*p* < 0.001) inactives, as well as in Strava actives (*p* = 0.025). And in corrected calf girth, a significant increase was recorded in the inactive Pacer (*p* = 0.024) and active Strava (*p* = 0.012). Only a significant decrease in fat mass (*p* = 0.011) and sum of 3 skinfolds (*p* < 0.001) was recorded in the inactive Pacer participants. In contrast, muscle mass increased significantly in all groups analyzed (*p* < 0.001–0.049). Regarding fitness tests, a significant increase in V02 max was found in Pacer inactive (*p* = 0.001) and Strava active (*p* < 0.001). Regarding the handgrip, there was an increase in the active and inactive Pacer (*p* < 0.001), as well as in the active Strava (*p* = 0.010). In curl-up, there was a significant increase in performance in those active and inactive on Strava (*p* < 0.001) and in those inactive on MapMyWalk (*p* = 0.014). And, in push-up, there was a significant improvement in those active (*p* = 0.013) and inactive (*p* = 0.028) from Pokémon Go. Despite the results obtained, Table 5 shows that the analysis of the change between the different applications did not show significant differences in any of the analyzed variables.

**Table 4:** Intra-group differences in the study variables in the groups of active and inactive adolescents according to the app used.

Variable	Group	Pokémon Go			Strava			Pacer			MapMyWalk		
		Diff.	η^2^	*p*-value	Diff.	η^2^	*p*-value	Diff	η^2^	*p*-value	Diff.	η^2^	*p*-value
Physical activity	Inactives	−0.33 ± 0.01	0.027	0.001	−0.38 ± 0.08	0.051	<0.001	−0.28 ± 0.09	0.026	0.001	−0.21 ± 0.10	0.012	0.032
	Actives	−0.01 ± 0.10	0.000	0.917	0.04 ± 0.10	0.001	0.648	0.12 ± 0.11	0.003	0.279	−0.09 ± 0.13	0.001	0.457
AMD	Inactives	−0.48 ± 0.48	0.003	0.311	−0.05 ± 0.39	0.000	0.905	0.43 ± 0.42	0.003	0.305	−0.21 ± 0.48	0.000	0.664
	Actives	1.03 ± 0.47	0.012	0.058	−0.42 ± 0.36	0.002	0.362	−0.26 ± 0.53	0.001	0.625	−0.33 ± 0.60	0.001	0.581
Body mass	Inactives	−0.56 ± 0.35	0.007	0.107	−1.25 ± 0.29	0.046	<0.001	−0.91 ± 0.31	0.022	0.003	−0.95 ± 0.35	0.019	0.007
	Actives	−1.04 ± 0.35	0.023	0.003	−1.33 ± 0.34	0.039	<0.001	−0.19 ± 0.39	0.001	0.629	−0.68 ± 0.44	0.006	0.125
Height (cm)	Inactives	−0.59 ± 0.30	0.010	0.042	−0.45 ± 0.26	0.008	0.049	−1.08 ± 0.27	0.041	<0.001	−0.47 ± 0.30	0.006	0.049
	Actives	−0.90 ± 0.30	0.023	0.003	−1.18 ± 0.29	0.041	<0.001	−0.77 ± 0.34	0.013	0.025	−0.39 ± 0.38	0.003	0.105
BMI	Inactives	0.04 ± 0.13	0.000	0.785	−0.34 ± 0.11	0.023	0.053	−0.09 ± 0.12	0.001	0.467	−0.20 ± 0.13	0.006	0.133
	Actives	−0.17 ± 0.13	0.004	0.195	−0.18 ± 0.13	0.005	0.153	0.32 ± 0.15	0.012	0.062	−0.16 ± 0.17	0.002	0.348
Waist-height	Inactives	−0.00 ± 0.00	0.000	0.830	−0.00 ± 0.00	0.001	0.529	−0.00 ± 0.00	0.011	0.250	−0.00 ± 0.00	0.001	0.551
	Actives	−0.00 ± 0.00	0.002	0.377	−0.00 ± 0.00	0.000	0.910	−0.00 ± 0.00	0.005	0.175	−0.00 ± 0.00	0.004	0.235
Corrected arm girth	Inactives	−0.29 ± 0.18	0.007	0.108	−0.24 ± 0.15	0.007	0.116	−0.42 ± 0.16	0.018	0.009	−0.46 ± 0.18	0.017	0.011
	Actives	−0.60 ± 0.18	0.029	<0.001	−0.48 ± 0.17	0.020	0.006	−0.33 ± 0.20	0.007	0.106	−0.66 ± 0.23	0.021	0.004
Corrected thigh girth	Inactives	−0.81 ± 0.42	0.010	0.055	−1.02 ± 0.36	0.021	0.004	−1.30 ± 0.38	0.030	<0.001	−0.57 ± 0.42	0.005	0.181
	Actives	−0.81 ± 0.42	0.010	0.052	−0.92 ± 0.41	0.013	0.025	−0.85 ± 0.47	0.008	0.073	−0.98 ± 0.54	0.009	0.068
Corrected calf girth	Inactives	−0.53 ± 0.35	0.006	0.131	0.53 ± 0.29	0.008	0.072	−0.71 ± 0.31	0.013	0.024	−0.40 ± 0.35	0.003	0.254
	Actives	−0.46 ± 0.34	0.005	0.182	−0.72 ± 0.34	0.005	0.012	−0.50 ± 0.39	0.004	0.199	−0.56 ± 0.44	0.004	0.206
Fat mass (%)	Inactives	0.37 ± 0.63	0.001	0.553	0.10 ± 0.53	0.000	0.846	1.44 ± 0.57	0.017	0.011	0.00 ± 0.63	0.000	0.996
	Actives	1.19 ± 0.62	0.010	0.056	0.20 ± 0.61	0.000	0.745	0.28 ± 0.71	0.000	0.695	1.50 ± 0.80	0.009	0.062
Sum of 3 skinfolds	Inactives	1.45 ± 1.66	0.002	0.384	0.93 ± 1.39	0.001	0.506	5.03 ± 1.49	0.029	<0.001	−0.63 ± 1.66	0.000	0.705
	Actives	3.18 ± 1.63	0.010	0.052	0.09 ± 1.60	0.000	0.958	0.92 ± 1.86	0.001	0.620	3.92 ± 2.10	0.009	0.063
Muscle mass (kg)	Inactives	−0.68 ± 0.25	0.020	0.006	−0.41 ± 0.21	0.010	0.047	−0.89 ± 0.22	0.041	<0.001	−0.48 ± 0.25	0.010	0.049
	Actives	−0.79 ± 0.25	0.026	0.001	−0.86 ± 0.24	0.034	<0.001	−0.65 ± 0.28	0.014	0.019	−0.90 ± 0.31	0.022	0.004
Vo2 Max	Inactives	−0.91 ± 0.57	0.007	0.113	−0.79 ± 0.47	0.008	0.093	−1.71 ± 0.52	0.031	0.001	−0.14 ± 0.58	0.000	0.804
	Actives	−0.98 ± 0.57	0.009	0.089	−2.13 ± 0.53	0.045	<0.001	−0.85 ± 0.64	0.005	0.182	−0.35 ± 0.75	0.001	0.642
Handgrip right hand	Inactives	−0.40 ± 0.84	0.001	0.633	−0.59 ± 0.69	0.002	0.395	−2.63 ± 0.74	0.031	<0.001	−1.39 ± 0.84	0.007	0.099
	Actives	−0.09 ± 0.82	0.000	0.913	−2.10 ± 0.81	0.017	0.010	−3.65 ± 0.94	0.037	<0.001	−1.32 ± 1.06	0.004	0.214
Handgrip left hand	Inactives	−0.41 ± 0.66	0.001	0.536	−0.20 ± 0.55	0.000	0.717	−1.82 ± 0.59	0.024	0.002	−1.08 ± 0.66	0.007	0.103
	Actives	−0.53 ± 0.65	0.002	0.414	−0.46 ± 0.64	0.001	0.472	−1.03 ± 0.75	0.005	0.167	−1.10 ± 0.84	0.004	0.192
CMJ	Inactives	−1.21 ± 1.49	0.002	0.415	−0.59 ± 1.22	0.001	0.629	−0.61 ± 1.32	0.001	0.646	0.45 ± 1.49	0.000	0.762
	Actives	−1.90 ± 1.46	0.004	0.195	−2.21 ± 1.44	0.006	0.126	−2.59 ± 1.67	0.006	0.123	−2.99 ± 1.89	0.006	0.115
Curl Up	Inactives	0.28 ± 1.95	0.000	0.888	−5.02 ± 1.62	0.060	<0.001	−1.31 ± 1.75	0.001	0.457	−4.93 ± 1.99	0.016	0.014
	Actives	−1.80 ± 1.92	0.002	0.349	−6.29 ± 1.89	0.059	<0.001	−2.35 ± 2.19	0.003	0.285	−0.56 ± 2.48	0.000	0.823
Push Up	Inactives	−2.62 ± 1.19	0.013	0.028	−0.98 ± 1.00	0.003	0.328	−1.03 ± 1.08	0.002	0.341	−1.21 ± 1.21	0.003	0.315
	Actives	−2.90 ± 1.17	0.016	0.013	−1.68 ± 1.15	0.006	0.144	−1.52 ± 1.33	0.003	0.254	−1.72 ± 1.50	0.003	0.253

BMI, body mass index; Sum of 3 skinfolds, summation of 3 skinfolds; Vo2 max, maximal oxygen consumption; CMJ, countermovement jump.

**Table 5:** Differences in the change between the pre and posttest in the study variables according to the app used.

Variables	Group	PokémonGo-Strava		PokémonGo-Pacer		PokémonGo-MapMyWalk		Strava-Pacer		Strava-MapMyWalk		Pacer-MapMyWalk	
		Mean Diff.	*p*-value	Mean Diff.	*p*-value	Mean Diff.	*p*-value	Mean Diff.	*p*-value	Mean Diff.	*p*-value	Mean Diff.	*p*-value
Physical activity	Inactives	0.11 ± 0.12	1.000	0.04 ± 0.12	1.000	0.00 ± 0.13	1.000	−0.07 ± 0.11	1.000	−0.11 ± 0.12	1.000	−0.04 ± 0.12	1.000
	Actives	−0.11 ± 0.13	1.000	−0.09 ± 0.14	1.000	0.02 ± 0.15	1.000	0.01 ± 0.13	1.000	0.13 ± 0.15	1.000	0.11 ± 0.16	1.000
AMD	Inactives	−0.51 ± 0.56	1.000	−1.04 ± 0.58	0.735	−0.30 ± 0.61	1.000	−0.53 ± 0.53	1.000	0.21 ± 0.56	1.000	0.74 ± 0.59	1.000
	Actives	0.55 ± 0.59	1.000	0.41 ± 0.65	1.000	0.65 ± 0.71	1.000	−0.15 ± 0.62	1.000	0.10 ± 0.69	1.000	0.25 ± 0.74	1.000
Body mass	Inactives	0.58 ± 0.44	1.000	−0.11 ± 0.46	1.000	0.43 ± 0.49	1.000	−0.69 ± 0.42	1.000	−0.15 ± 0.45	1.000	0.54 ± 0.47	1.000
	Actives	0.42 ± 0.47	1.000	−0.86 ± 0.52	0.971	−0.14 ± 0.57	1.000	−1.28 ± 0.50	0.105	−0.56 ± 0.55	1.000	0.72 ± 0.59	1.000
Height (cm)	Inactives	−0.14 ± 0.34	1.000	0.05 ± 0.36	1.000	−0.06 ± 0.38	1.000	0.19 ± 0.33	1.000	0.08 ± 0.35	1.000	−0.11 ± 0.36	1.000
	Actives	0.27 ± 0.36	1.000	−0.28 ± 0.40	1.000	−0.61 ± 0.44	1.000	−0.55 ± 0.38	1.000	−0.88 ± 0.43	1.000	−0.33 ± 0.45	1.000
BMI	Inactives	0.34 ± 0.19	0.645	0.07 ± 0.19	1.000	0.23 ± 0.20	1.000	−0.28 ± 0.18	1.000	−0.12 ± 0.19	1.000	0.16 ± 0.20	1.000
	Actives	0.06 ± 0.20	1.000	−0.48 ± 0.22	0.267	0.07 ± 0.24	1.000	−0.54 ± 0.21	0.100	0.01 ± 0.23	1.000	0.55 ± 0.25	0.271
Waist-height	Inactives	−0.00 ± 0.00	1.000	−0.01 ± 0.00	0.328	−0.00 ± 0.00	1.000	−0.01 ± 0.00	0.334	−0.00 ± 0.00	1.000	0.01 ± 0.00	1.000
	Actives	0.00 ± 0.00	1.000	−0.00 ± 0.00	1.000	−0.00 ± 0.01	1.000	−0.01 ± 0.00	1.000	−0.00 ± 0.00	1.000	0.00 ± 0.01	1.000
Corrected arm girth	Inactives	−0.05 ± 0.25	1.000	−0.00 ± 0.27	1.000	0.10 ± 0.28	1.000	0.04 ± 0.24	1.000	0.15 ± 0.26	1.000	0.11 ± 0.27	1.000
	Actives	0.01 ± 0.27	1.000	−0.11 ± 0.30	1.000	0.32 ± 0.33	1.000	−0.12 ± 0.28	1.000	0.31 ± 0.32	1.000	0.43 ± 0.34	1.000
Corrected thigh girth	Inactives	0.01 ± 0.59	1.000	0.42 ± 0.62	1.000	0.09 ± 0.65	1.000	0.41 ± 0.56	1.000	−0.10 ± 0.60	1.000	−0.51 ± 0.62	1.000
	Actives	−0.05 ± 0.63	1.000	0.02 ± 0.69	1.000	0.63 ± 0.76	1.000	0.07 ± 0.66	1.000	0.68 ± 0.73	1.000	0.62 ± 0.78	1.000
Corrected calf girth	Inactives	−1.13 ± 0.49	0.224	0.08 ± 0.51	1.000	−0.20 ± 0.54	1.000	1.21 ± 0.47	0.101	0.93 ± 0.50	0.621	−0.28 ± 0.52	1.000
	Actives	0.07 ± 0.52	1.000	0.10 ± 0.57	1.000	0.38 ± 0.63	1.000	0.03 ± 0.55	1.000	0.31 ± 0.61	1.000	0.28 ± 0.65	1.000
Fat mass (%)	Inactives	0.06 ± 0.87	1.000	−1.21 ± 0.91	1.000	0.37 ± 0.96	1.000	−1.27 ± 0.83	1.000	0.31 ± 0.88	1.000	1.58 ± 0.92	0.865
	Actives	1.55 ± 0.93	0.961	1.01 ± 1.01	1.000	−0.77 ± 1.12	1.000	−0.54 ± 0.97	1.000	−2.32 ± 1.08	0.332	−1.78 ± 1.16	1.000
Sum of 3 skinfolds	Inactives	0.73 ± 2.31	1.000	−3.65 ± 2.41	1.000	2.26 ± 2.54	1.000	−4.38 ± 2.19	0.467	1.53 ± 2.33	1.000	5.91 ± 2.44	0.157
	Actives	4.52 ± 2.45	0.665	2.75 ± 2.68	1.000	−2.17 ± 2.96	1.000	−1.77 ± 2.58	1.000	−6.69 ± 2.87	0.201	−4.93 ± 3.06	1.000
Muscle mass (kg)	Inactives	−0.36 ± 0.34	1.000	0.08 ± 0.36	1.000	−0.19 ± 0.38	1.000	0.44 ± 0.33	1.000	0.18 ± 0.35	1.000	−0.26 ± 0.36	1.000
	Actives	0.05 ± 0.36	1.000	−0.09 ± 0.40	1.000	0.50 ± 0.44	1.000	−0.14 ± 0.38	1.000	0.45 ± 0.43	1.000	0.59 ± 0.45	1.000
Vo2 Max	Inactives	0.02 ± 0.74	1.000	0.80 ± 0.77	1.000	−0.76 ± 0.81	1.000	0.79 ± 0.70	1.000	−0.78 ± 0.75	1.000	−1.57 ± 0.78	0.451
	Actives	1.15 ± 0.78	1.000	−0.13 ± 0.86	1.000	−0.63 ± 0.95	1.000	−1.28 ± 0.82	1.000	−1.78 ± 0.92	0.528	−0.50 ± 0.98	0.979
Handgrip right hand	Inactives	1.31 ± 0.91	1.000	1.55 ± 0.95	1.000	0.23 ± 1.00	1.000	0.23 ± 0.86	1.000	−1.09 ± 0.92	1.000	−1.32 ± 0.96	1.000
	Actives	2.21 ± 0.97	0.228	3.04 ± 1.06	0.079	0.92 ± 1.17	1.000	1.32 ± 1.02	1.000	−1.30 ± 1.13	1.000	−2.62 ± 1.21	0.307
Handgrip left hand	Inactives	0.46 ± 0.71	1.000	0.62 ± 0.74	1.000	−0.03 ± 0.78	1.000	0.17 ± 0.67	1.000	−0.48 ± 0.72	1.000	−0.65 ± 0.75	1.000
	Actives	0.43 ± 0.75	1.000	0.86 ± 0.82	1.000	0.17 ± 0.91	1.000	0.43 ± 0.79	1.000	−0.25 ± 0.88	1.000	−0.69 ± 0.94	1.000
CMJ	Inactives	−0.31 ± 1.14	1.000	−0.08 ± 1.19	1.000	−0.25 ± 1.25	1.000	0.24 ± 1.08	1.000	0.06 ± 1.15	1.000	−0.18 ± 1.20	1.000
	Actives	1.26 ± 1.21	1.000	0.43 ± 1.32	1.000	0.36 ± 1.46	1.000	−0.83 ± 1.27	1.000	−0.90 ± 1.42	1.000	−0.07 ± 1.51	1.000
Curl Up	Inactives	5.05 ± 2.67	0.098	2.25 ± 2.79	1.000	4.57 ± 2.94	1.000	−5.80 ± 2.54	0.230	−3.48 ± 2.70	1.000	2.32 ± 2.82	1.000
	Actives	5.21 ± 2.84	0.101	1.39 ± 3.11	1.000	−1.05 ± 3.43	1.000	−6.82 ± 2.99	0.230	−9.27 ± 3.32	0.055	−2.45 ± 3.55	1.000
Push Up	Inactives	−1.36 ± 1.57	1.000	−1.53 ± 1.64	1.000	−0.70 ± 1.73	1.000	−0.17 ± 1.49	1.000	0.66 ± 1.59	1.000	0.83 ± 1.66	1.000
	Actives	−2.01 ± 1.67	1.000	−2.48 ± 1.83	1.000	−1.61 ± 2.02	1.000	−0.47 ± 1.76	1.000	0.40 ± 1.95	1.000	0.87 ± 2.09	1.000

BMI, body mass index; Sum of 3 skinfolds, summation of 3 skinfolds; Vo2 max, maximal oxygen consumption; CMJ, countermovement jump.

## 4 Discussion

### 4.1 Main results of the present research

The first objective of the present research was to analyze the differences in the level of physical activity, AMD, anthropometry, body composition, and physical fitness of active and inactive adolescents achieved by a 10-week program of after-school use of step tracker mobile apps promoted from the physical education subject. The results showed that there was no difference in the distance traveled by the adolescents with the use of the mobile application depending on whether they were active or inactive, nor depending on the app used. Regarding the perceived level of physical activity, there were significant increases in both inactive groups (EG and CG) and in the active CG, while in AMD there were no significant differences in either group between the pre- and post-test. In the kinanthropometric and body composition variables, the results showed significant increases in body mass, height, BMI, corrected girths and muscle mass in all the groups. Furthermore, in active and inactive adolescents, a decrease in fat mass and in the sum of 3 skinfolds was observed in the EGs. Regarding the physical fitness variables, the EGs of active and inactive adolescents showed improved performance in all the physical fitness tests, with the exception of the CMJ in the inactive group.

The second objective of the research was to determine the influence of gender, maturity, the app used, and the number of steps taken with the mobile apps on the study variables. Gender showed a significant effect on the kinanthropometric and body composition variables, physical fitness test and physical activity level of the active and inactive adolescents in CG and EG. The maturity status seemed to influence the body mass and height, the muscle mass variables and most physical fitness test in all the groups. According to the app used, this covariate influenced the level of physical activity of inactive adolescents, as well as the body mass, VO2 max and curl up of active and inactive adolescents. And the covariate distance covered with the app showed significant influence in the physical activity, body mass, height, BMI, corrected arm girth, corrected thigh girth, sum of 3 skinfolds, muscle mass and all the physical fitness test, except CMJ, on the inactive adolescents; as well as in the body mass, height, corrected arm girth, corrected thigh girth, corrected calf girth, muscle mass and all the physical fitness test on the active adolescents.

Despite the results obtained, the analysis of the change in the differences found in the study variables in the EG and CG in both the active and inactive groups showed that only the change in BMI was significantly greater in the CG than in the EG, with a significant influence of the covariates gender and maturity; and that the changes in CMJ were greater in the EG than in the CG, with a significant influence of the covariate maturity. It should also be noted that, although there were differences between the changes caused by the four apps when analyzing pre-post intra-group differences, there were no inter-group differences in the changes generated by the different apps.

### 4.2 Explanation of the changes found in the study variables

According to the kinanthropometric and body composition variables, one of the main findings of the present investigation was the decrease in fat mass and in the sum of 3 skinfolds in active and inactive adolescents of the EGs, without significant changes in active and inactive adolescents of the CGs. This could lead to consider that the interventions with step tracker mobile apps can slightly reduce the accumulation of fat mass in the adolescent population, regardless of the initial level of physical activity of the adolescents. Thus, the increased physical activity performed by the adolescents in the EG would increase their energy expenditure, which would favor the loss of fat mass, as observed in previous research (94). However, it should be noted that the active adolescents in the EG did not report a significant increase in the physical activity performed. This could be due to the fact that when recording this variable by means of a self-completed questionnaire, these students did not perceive that going for a walk three times a week increased the daily physical activity performed.

In terms of muscle mass variables, both active and inactive EG and CG adolescents showed significant increases after the intervention, which leads to consider that the changes were not solely due to the intervention performed with the step tracker mobile apps. The fact that participants used aerobic applications where no strength-endurance training was performed leads one to believe that changes in muscle mass were not generated by the intervention (95). Therefore, a possible explanation for these results could be that the maturation process in which adolescents are immersed was the cause of the changes in muscle mass variables, which explains why changes in muscle mass variables also occurred in the CGs (96). Puberty is a key stage in adolescent musculoskeletal development, and is characterized by increases in the accumulation of sex steroid hormones such as testosterone or growth hormone (96–98). This increase in hormone concentration is highly correlated with increased muscle mass, as well as increased strength production (99–101).

Similarly, the observed changes in height and body mass in all the groups could be due to the maturational process (102). Previous research has shown that the maximum peak growth (APHV) occurs in girls between the ages of 11.4 and 12.2, and in boys between 13.8 and 14.4 years of age increasing in height during these ages at a rate of 9–10.3 cm per year (60, 103, 104). Body mass is also affected during this stage, increasing by 8.3 kg per year in girls and 9 kg per year in boys (105, 106). Furthermore, changes in height and body mass would be the cause of the changes in BMI, which would explain why this variable was modified in all groups. However, despite being a widely used index in previous scientific literature it does not allow distinguishing whether the changes are due to changes in fat mass or muscle mass (107, 108).

Therefore, based on the results concerning kinanthropometric and body composition variables, it seems that the 10-week intervention with step tracker mobile apps could generate changes only in fat variables. However, the changes in body mass, height, BMI, and muscle mass variables seem to be the result of changes caused by the maturation process in which adolescents are immersed. The results obtained in the present study support these assertions, since the inclusion of the covariate maturity showed influence on all the kinanthropometric and body composition variables, with the exception of fat mass and sum of 3 skinfolds.

Regarding the physical fitness variables, the EGs of active and inactive adolescents showed improved performance in all the physical fitness tests, with the exception of the CMJ in the inactive group. In this case, the covariate maturity was shown to be determinant in the physical fitness tests related to muscular strength (handgrip and push-ups), but not in those of cardiorespiratory capacity or curl-up in which the distance covered with the app were more relevant. A possible explanation for these results could be that walking programs favor the strength-endurance of the abdominal muscles, as walking activates the core-stabilizing muscles, and could improve it after 10 weeks of intervention (109, 110). In terms of cardiorespiratory capacity, the use of aerobic applications based on walking would help increase performance in this test in both active and inactive adolescents, by including in the intervention, a weekly increase in the volume of walking to end in a daily distance equivalent to what an active adolescent should do (57). Differences were found in CMJ performance in the group of active adolescents, but the absence of these differences in the group of inactive adolescents could be due to the fact that this test requires a correct technical execution, which is present in many sports when performing vertical jumps (111, 112). This could indicate that in athletes with previous experience with this type of test, the improvements in vertical jump performance after the mobile app-based intervention were due to an improvement in lower limb strength (61). However, in inactive subjects, this improvement could be diluted by the lack of proper technique during the execution of the test by the participants.

The fact that all groups in the study improved handgrip and push-up performance, together with the fact that the covariate maturity showed influence in these tests, and the fact that mobile apps were not included for strength work, lead us to consider that the changes in these variables would be more related to the increase in muscle mass and strength as a result of the maturation process, than to the 10-week intervention (113). Therefore, another finding of relevance of the present investigation was that the fitness variables that could be most affected by the 10-week intervention were VO2 max and curl-up, while the rest of the variables seem to be more affected by the maturational process and associated changes in muscle mass.

It is also worth mentioning that the level of physical activity only showed significant changes in inactive adolescents. This could be because the perception of active adolescents, regardless of the volume of training completed, is that walking is not a sporting activity in the way that practicing a particular sport might be, so their perception is that the use of apps does not increase the physical activity performed. However, in the group of inactive adolescents, whose level of practice was lower, the inclusion of walking three times a week would be perceived as a substantial increase in the physical activity practiced.

Similarly, the absence of differences in AMD in either group is a relevant finding. The fact that the selected step tracker mobile apps did not include any type of nutritional guidelines could explain the results found, as previous studies using mobile nutrition applications have shown them to be effective in controlling intake, and in achieving changes in the anthropometry and body composition of adolescents (57, 114).

However, despite the results obtained, it should be noted that the results of the analysis of the change observed between EG and CG adolescents were only significant in BMI and CMJ for active participants, and the effect sizes of these changes were small in all the variables analyzed. More specifically, there was a greater body mass gain in the active CG, and a greater CMJ gain in the active EG. Therefore, the evolution of most variables showed no significant change between the EG and the CG. On the one hand, this could be because longer interventions may be necessary to obtain differences in the changes between EG and CG (115). On the other hand, intensity, as opposed to distance, could be a stronger differentiating factor for generating changes in some of these variables (58, 116). Therefore, although some of the changes achieved during the research in the EG seem to be the result of the 10-week intervention with mobile applications, future research is needed to determine the real potential of this type of tool to increase physical activity and achieve substantial changes in kinanthropometric, body composition and physical fitness variables in the adolescent population.

### 4.3 Importance of the covariates included in the present research

The results obtained highlight the importance of the covariates included in the present study. The covariate gender seems to influence the level of physical activity, kinanthropometric and body composition variables, as well as physical fitness variables. Previous research has shown that the practice of physical activity of adolescent males tends to be higher than that of adolescent females (117). The results of the present research could indicate that step tracker mobile apps are a good strategy to increase physical activity practice in active and inactive females. These results are in line with previous research where it was observed that females used physical activity apps to a higher extent than males when their use was promoted in physical education classes (118). Regarding anthropometry and body composition, differences were observed in the muscle mass of all the groups analyzed, and in the fat mass of the EG. These results are consistent with those found in previous research, in which the muscle mass of adolescent males was higher than that of adolescent females (119). It is true that during the maturation process, steroid hormones increase in both males and females, but the accumulation is much greater in males, which generates greater increases in muscle mass (59, 120–122). In contrast, there is a greater accumulation of fat mass in females during puberty, which would be influencing the effects of the intervention program (59, 120, 121). Similarly, differences were significant in the majority of the physical fitness variables in the EG of active and inactive adolescents. It should also be noted that in the physical fitness tests related to body strength and cardiorespiratory capacity, the differences were significant according to gender in all groups. The greater development of muscle mass and strength of males with respect to females during puberty, as well as the greater cardiorespiratory capacity shown in previous research, could be the explanation for the results found (123). Another explanation could be that males generally practice more physical activity than females, so it is possible that this is the factor that causes a higher performance in all the fitness test in males (109).

The maturational process in which adolescents are immersed plays a very relevant role in the results of this research. As it has been observed, most of the changes in the variables of muscle mass, as well as in body mass, height and BMI are influenced by maturation. Similarly, the physical fitness variables related to strength, mainly handgrip and push-ups present a similar influence. This is due to hormonal changes and muscle mass development that occur during puberty (122). However, the fact that the changes in the level of physical activity, fat mass variables, VO2 max and curl up were not affected by this covariate, suggests that the intervention generates changes in these variables.

A significant finding was that the app used has been shown to influence the level of physical activity of inactive adolescents, as well as kinanthropometric and body composition, and physical fitness variables for both active and inactive adolescents. Specifically, the results showed differences in physical activity level, body mass, height, corrected perimeters, fat mass, sum of 3 skinfolds, muscle mass, V02 max, handgrip, curl up and push up when analyzing each of the mobile apps individually. However, the change in these variables between the different mobile apps was not significant, so there were no different effects between apps. These results show that the applications, even if they have different techniques for behavior change (84), can have similar effects. This is relevant because step-counting apps, such as Strava, Pacer and MapMyWalk, as well as gamified apps, such as Pokémon Go, can be effective with both active and inactive adolescents. These results are similar with previous research in which differences were seen in the study variables, mainly body mass, handgrip and curl up, depending on the application used (61), but bring the great novelty that the change produced between apps in these variables is not significant. Furthermore, the fact that the selected application shows different effects on the increase in physical activity depending on the level of physical activity is very relevant. That said, it would be important for future research to analyze the differences between interventions delivered with different apps in the longer term, to see if the fact that some of them are gamified may have an influence on the psychological variables that lead to greater use of them in the longer term, as has been seen in research in other fields (124, 125).

And, regarding the covariate distance covered with the use of the apps, the results showed a significant influence on the kinanthropometric and body composition variables both in active and inactive adolescents. This could indicate that the greater the distance covered with the application, the greater the changes achieved in the anthropometry and body composition of adolescents, regardless of whether or not they regularly practiced physical activity. These results are of great relevance, as they would indicate that the completion of a 10-week aerobic training program using step tracker mobile apps for its quantification would be effective in producing improvements in anthropometry and body composition in this population, at least for the reduction in fat mass, which would follow the line of previous research that used step tracker mobile apps (58). Similarly, the distance traveled with the use of the step tracker mobile apps showed an influence on all physical fitness variables, regardless of whether the adolescents were active or inactive before starting the research. It is logical to think that regardless of the previous level of practice, the improvements would be greater in adolescents who completed a longer distance with the use of step tracker mobile apps, as they would be more active, allowing them to obtain a greater physical fitness, as shown in previous research (58).

### 4.4 Differences and similarities of the results found with respect to previous scientific evidence

The main contribution of the present investigation with respect to previous scientific evidence is that active and inactive adolescents after the use of mobile applications for 10 weeks could show changes in fat mass, cardiorespiratory capacity and the strength-endurance of the abdominal muscles. It is true that previous research had shown the benefits of physical activity interventions, and more specifically of interventions with mobile applications, on these variables in the adolescent population (58). In this regard, previous research have shown a decrease in fat variables, as well as an improvement in performance in physical fitness tests, such as CMJ, curl-up, push-up or handgrip, after the interventions (46, 61). This is similar to the results obtained in the present research; however, the main contribution of this study and which had not been observed in any previous study is that the results are equally significant for previously active and inactive adolescents, with no significant differences between these groups, this study being novel in that sense. In addition, the fact that curl-up and cardiorespiratory capacity were not influenced by maturity is also new to this study and could indicate the effects of the intervention on these variables.

The improvement in other physical fitness variables as handgrip and push-up after intervention with step-tracking mobile apps also follows the line of previous research (61). However, this study provides two main novelties. The first is that both active and inactive adolescents belonging to the GE and the CG showed benefits in these tests. The second is that the maturity covariate was shown to significantly influence these variables. Therefore, this is the first study that shows the effect that maturity could play in the changes obtained in tests related to upper limb strength in this type of intervention. It is logical to think of the effect of maturity since this intervention did not include upper limb strength work, but in previous investigations this argument was used to justify the changes and in none of them was the influence statistically demonstrated.

Another novelty of the present study is that the increases in the level of physical activity occurred only in the group of inactive adolescents. Previous studies have shown that the implementation of step-tracker app-based interventions could increase self-perceived physical activity in adolescents (126). However, no previous study had examined the influence of adolescents’ previous activity level on this change. The present novel study is novel in this respect. Furthermore, the absence of differences in AMD after intervention with step tracker mobile apps had been also observed in previous research (115).

In addition, the inclusion of the covariates gender, maturity, app used and distance traveled with the app and their effect on the study variables allows us to learn more about the changes that can actually be caused by the 10-week intervention with step tracker mobile apps. Previous studies have suggested that the effects of step-tracker intervention could depend on gender, the app used or the distance traveled with it (61, 118). However, in the case of the app used, the changes in the study variables when comparing between the different mobile applications were not significant, so there was no greater effect of one app on the others in either the active or inactive group. For its part, it has been shown that maturation could have a significant influence on the effects of physical exercise on anthropometric variables, body composition or physical fitness (120). However, this is the first research to analyze the effects of these factors according to whether the adolescents were active or inactive.

According to the results obtained in the present investigation, H1 in which indicated that the use of mobile step-tracking apps will be more effective on the study variables in inactive adolescents, since their level of physical activity is lower than that of active ones, can be partially rejected. This is because the changes occurred in both active and inactive adolescents, with the exception of the level of physical activity, in which only inactive adolescents perceived a greater improvement after the intervention, and the CMJ test, where significant changes were found only in the active adolescents. Regarding H2 in which indicated that gender, maturity, app used and the number of steps taken with the mobile apps will influence the changes produced in the study variables after the use of the mobile apps, it can be accepted. This is because all four covariates were shown to influence the study variables, mainly gender and maturity, although in the case of the app used there were no significant differences when considering the change produced by each of the apps.

### 4.5 Limitations and future directions

The present investigation is not without limitations. The sample was selected by convenience, by choosing the compulsory secondary schools with the largest number of adolescents in the geographical area of study. The level of physical activity was assessed using the PAQ-A questionnaire, which is a subjective measure of physical activity, which could influence the results obtained. It is also important to note that this questionnaire only records the physical activity of the last week, which might not give an overall picture of the subject’s level of physical activity. In addition, the PAQ-A classifies subjects as active or inactive, without providing an intermediate range between the two conditions of physical activity. In addition, although the results of the KIDMED questionnaire did not show changes in adolescent AMD, other instruments should be used to assess adolescent caloric intake, as it could significantly influence changes in the anthropometry and body composition in this population.

In view of the limitations present in the research, the following lines of future research are proposed: (a) it would be important to complement the subjective information on perceived physical activity obtained by means of questionnaires with an objective measure. This could be carried out by accelerometry, or by means of wearable devices that allow the collection of data on physical activity throughout the day; (b) the use of mobile applications that include strength-endurance exercises would allow to know whether the effect of the maturational process is the cause of changes in muscle mass variables and fitness tests, or whether these are also modified by appropriate training; (c) interventions that include nutritional and physical activity guidelines are more effective than those that isolate one of these behaviors (97). Considering the absence of changes in AMD with the present intervention, future studies should use mobile step-tracking apps that track both physical activity and nutrition, or that include recommendations in both areas, as the results could be superior to those found in the present investigation; (d) future research should address the effect of the step-tracker apps on the results found, as it could be very relevant to decrease the time of physical inactivity in adolescents; (e) should also be examined in depth as to whether the distance traveled may be a more important determinant factor in an inactive or active group; and (f) this is the first study using the mobile apps and dividing the results according to the previous physical activity level of the adolescents, so the results should be taken with caution and future research is needed to corroborate them.

### 4.6 Practical implication

The following practical applications are derived from this research: (a) the step tracker mobile apps can be a very useful tool to be used in physical education classes, or promoted in the field of health, to increase the level of physical activity of inactive adolescents; (b) the use of mobile app step trackers can be a useful tool to favor the perception of competence, autonomy and social relationships in relation to physical activity. This could increase the satisfaction of the basic psychological needs of adolescents, which could be especially important among inactive adolescents. Thus, the use of mobile apps for the promotion of physical activity could lead to an improvement in the basic psychological needs of adolescents and, therefore, to greater adherence to the practice of physical activity; (c) the improvement in the fat variables after the intervention, both in the group of active and inactive adolescents, leads to consider these interventions as effective in improving body composition, which is decisive due to the increase in the rate of overweight and obesity in the adolescent population, especially among inactive adolescents, and could be a tool of great interest for the promotion of public health; (d) the use of rewards from the physical education class seems to be a useful strategy to increase participation and gain benefits from the intervention. This could be due to the fact that this external factor influences external regulation, which would affect adolescents’ motivation. Therefore, if the use of apps is promoted from the physical education subject with an increase in the grade, initial adherence to the program may benefit; and (e) the present study demonstrates the benefits of the use of mobile applications in both active and inactive adolescents, which is fundamental to combat the negative perception that currently exists toward the use of mobile devices in this population. This leads to the view that it is not so much the time spent using the devices that is really harmful, but the apps used.

## 5 Conclusion

Regardless of the initial level of physical activity, the use of step tracker mobile apps in out-of-school hours, promoted from the subject of physical education, resulted in significant improvements in fat variables and in cardiorespiratory capacity and curl-up performance of adolescents aged 12–16 years old. The evolution of the rest of the kinanthropometric and body composition variables, as well as the physical fitness variables, seem to be more strongly influenced by gender and maturity status than by the physical exercise intervention. It should be noted that although both active and inactive adolescents in the EG used the step tracker mobile apps during the intervention period, only adolescents who were inactive before the start of the study perceived improvements in their level of physical activity. In addition, the app used and the distance covered with the apps seem to be influential in the results. Therefore, mobile step tracking apps may be a useful alternative to obtain improvements in fat variables and in some of the physical fitness variables, but the results obtained should be taken with caution because it is the first research to be carried out dividing adolescents according to their previous level of physical activity.

## Data availability statement

The raw data supporting the conclusions of this article will be made available by the authors, without undue reservation.

## Ethics statement

The studies involving humans were approved by Institutional committee of the Catholic University of Murcia (code: CE022102). The studies were conducted in accordance with the local legislation and institutional requirements. Written informed consent for participation in this study was provided by the participants’ legal guardians/next of kin.

## Author contributions

NG-C: Formal analysis, Investigation, Writing – original draft, Writing – review & editing. AM-O: Conceptualization, Data curation, Formal analysis, Investigation, Methodology, Writing – original draft, Writing – review & editing. LM: Investigation, Writing – original draft, Writing – review & editing. LA-C: Conceptualization, Funding acquisition, Investigation, Project administration, Writing – original draft, Writing – review & editing. RV-C: Conceptualization, Data curation, Formal analysis, Funding acquisition, Investigation, Methodology, Project administration, Writing – original draft, Writing – review & editing.
